# Supplementary material for: Accuracy of AI-based binary classification for detecting malocclusion in the mixed dentition stage
Source: PLoS One. 2025 Dec 9;20(12):e0338713. doi: 10.1371/journal.pone.0338713 (PMC12688100; doi:10.1371/journal.pone.0338713)
Supplement: Attachment — Submitted filename: Response to Reviewers.docx [file pone.0338713.s001.docx]

**Response to Reviewers**

**Response to Academic editor:**

*Thank you for submitting your manuscript to PLOS ONE. After careful consideration, we feel that it has merit but does not fully meet PLOS ONE’s publication criteria as it currently stands. Therefore, we invite you to submit a revised version of the manuscript that addresses the points raised during the review process. The study question is quite interesting, however there are several aspects, especially methodological ones, to be considered. If you decide to resubmit an adjusted version, please incorporate the comments of the 3 reviewers.*

*Please include the following items when submitting your revised manuscript:*

- *A rebuttal letter that responds to each point raised by the academic editor and reviewer(s). You should upload this letter as a separate file labeled 'Response to Reviewers'.*
- *A marked-up copy of your manuscript that highlights changes made to the original version. You should upload this as a separate file labeled 'Revised Manuscript with Track Changes'.*
- *An unmarked version of your revised paper without tracked changes. You should upload this as a separate file labeled 'Manuscript'.*

*If you would like to make changes to your financial disclosure, please include your updated statement in your cover letter. Guidelines for resubmitting your figure files are available below the reviewer comments at the end of this letter.*

**RESPONSE:**

We sincerely appreciate the valuable and insightful comments of the editors and reviewers. We have revised and improved the manuscript as you have indicated. Below are our responses to the issues raised. We have also highlighted in the “Revised Manuscript with Track Changes” file the areas where we have updated the manuscript in response to the reviewers' comments.

*If applicable, we recommend that you deposit your laboratory protocols in protocols.io to enhance the reproducibility of your results. Protocols.io assigns your protocol its own identifier (DOI) so that it can be cited independently in the future. For instructions see:* [*https://journals.plos.org/plosone/s/submission-guidelines#loc-laboratory-protocols*](https://track.editorialmanager.com/CL0/https:%2F%2Fjournals.plos.org%2Fplosone%2Fs%2Fsubmission-guidelines%23loc-laboratory-protocols/1/010f01955add63ed-aee6f054-3f4c-4e0d-a73c-31e3d202f3a9-000000/yePOaEznMw-HkuYCtITHjZnBEwl3REJ7iJNmHyuPCzw=200)*. Additionally, PLOS ONE offers an option for publishing peer-reviewed Lab Protocol articles, which describe protocols hosted on protocols.io. Read more information on sharing protocols at* [*https://plos.org/protocols?utm_medium=editorial-email&utm_source=authorletters&utm_campaign=protocols*](https://track.editorialmanager.com/CL0/https:%2F%2Fplos.org%2Fprotocols%3Futm_medium=editorial-email%26utm_source=authorletters%26utm_campaign=protocols/1/010f01955add63ed-aee6f054-3f4c-4e0d-a73c-31e3d202f3a9-000000/M3XRP4bXXyV54W6TTnn7Ok4Uuwj1MN3Le3MHGx-GaN4=200)*.*

**RESPONSE:**

Thank you for recommending that we register our protocol on Protocols.io. We hope to publish the results of our study as soon as possible in a peer-reviewed open access journal. However, the protocol registration process is expected to take some time. First, we need to prepare the necessary documents for protocol registration on Protocols.io. After that, we must obtain approval from the Institutional Review Board (IRB) regarding the publication of the protocol. Therefore, we will revisit this matter once the manuscript has been accepted for publication.

*Journal Requirements:*

*When submitting your revision, we need you to address these additional requirements.*

*1.Please ensure that your manuscript meets PLOS ONE's style requirements, including those for file naming. The PLOS ONE style templates can be found at*

*https://journals.plos.org/plosone/s/file?id=wjVg/PLOSOne_formatting_sample_main_body.pdf and*

*https://journals.plos.org/plosone/s/file?id=ba62/PLOSOne_formatting_sample_title_authors_affiliations.pdf*

**RESPONSE:**

We have reviewed PLOS ONE's style requirements and have corrected font size, formatting, etc. We made a mistake with the font size in Level 1-3 Heading, so We corrected it to the correct size. We also have changed the format of the formula for the model's evaluation index, as noted on page 20. We hope that these revisions now adhere to the journal’s style requirements.

*2. Please note that PLOS ONE has specific guidelines on code sharing for submissions in which author-generated code underpins the findings in the manuscript. In these cases, we expect all author-generated code to be made available without restrictions upon publication of the work. Please review our guidelines at https://journals.plos.org/plosone/s/materials-and-software-sharing#loc-sharing-code and ensure that your code is shared in a way that follows best practice and facilitates reproducibility and reuse.*

**RESPONSE:**

This study utilizes the AI platform provided by Datarobot to create the algorithm, due to restrictions in the license agreement with Datarobot Inc, we are unable to share regarding the code of the algorithm. Therefore, we have added information about the algorithm as much as possible in the Method sections of the text. In the Method section, the subtopic “Modelling and valdation” on page 16 – 17, we have described information on the blue prints of each algorithm we have developed.

P17, lines 3 – P18, lines 2:

The following modeling was performed by Datarobot on autopilot. Questionnaire data and image data were automatically separated from the dataset, and feature extraction from the image data was performed by a pre-trained deep learning model of SqueezeNet, a type of CNN that had been pre-trained. Next, Datarobot used the binary data from questionnaire data and the extracted image features as explanatory variables to create a classification model for the presence or absence of overbite using the Elastic Net Classifier.

In the overjet classification model, before building the model using all data sets, Datatobot only used the feature values extracted by SqueezeNet, and calculated the predicted value for the presence or absence of overjet using the Elastic Net Classifier. Then, Datarobot used the predicted value itself as a new feature value, integrated it with the binary data from the questionnaire data, and constructed an algorithm for judging overjet using the Elastic Net Classifier.

In the crowding classification model, before building the model using all data sets, Datarobot used only the image features extracted by SqueezeNet as explanatory variables and calculated predictions for the presence or absence of crowding using the Elastic Net Classifier. Datatobot adopted the above prediction values themselves as new features. By feeding the binary data from the questionnaire and the prediction values into the SVM Classifier model as explanatory variables, we constructed a crowding classgfcation model.

*3. Please provide additional details regarding participant consent. In the ethics statement in the Methods and online submission information, please ensure that you have specified what type you obtained (for instance, written or verbal, and if verbal, how it was documented and witnessed). If your study included minors, state whether you obtained consent from parents or guardians. If the need for consent was waived by the ethics committee, please include this information.*

*Once you have amended this/these statement(s) in the Methods section of the manuscript, please add the same text to the “Ethics Statement” field of the submission form (via “Edit Submission”).*

**RESPONSE:**

We added more detail on how to provide participant consent. The revisions are as follows.

P10, lines 1–3:

When obtaining consent, a consent form was displayed on the web screen and buttons were provided for subjects to indicate whether they agreed or disagreed.

P10, lines 8–11:

Consent was obtained by placing a response button on the website. Parents or guardians were asked to indicate whether they had obtained consent from their child by pressing the button.

*4. Thank you for stating the following financial disclosure:*

*“This work was funded by Lion Corporation (*[*https://www.lion.co.jp/en/)*](https://www.lion.co.jp/en/))*.” Please state what role the funders took in the study. If the funders had no role, please state: "The funders had no role in study design, data collection and analysis, decision to publish, or preparation of the manuscript."*

*If this statement is not correct you must amend it as needed. Please include this amended Role of Funder statement in your cover letter; we will change the online submission form on your behalf.*

**RESPONSE:**

The funding organization (Lion Corporation) did not play any role in the study design, data collection and analysis, decision to publish, or preparation of the manuscript. It only provided financial support in the form of authors’ salaries and/or research materials. Therefore, we stated “The funders had no role in study design, data collection and analysis, decision to publish, or preparation of the manuscript.” in the original Funding Statement. We have also included the above information in the cover letter.

*5. Thank you for stating the following in the Competing Interests section:*

*“I have read the journal's policy and the authors of this manuscript have the following competing interests:*

*[The authors of this manuscript employed by LION Corporation. Recruitment of participants was performed by Macromill Inc. (Tokyo, Japan) under consignment from Lion Corporation.]”*

*We note that one or more of the authors are employed by a commercial company: LION Corporation*

*a. Please provide an amended Funding Statement declaring this commercial affiliation, as well as a statement regarding the Role of Funders in your study. If the funding organization did not play a role in the study design, data collection and analysis, decision to publish, or preparation of the manuscript and only provided financial support in the form of authors' salaries and/or research materials, please review your statements relating to the author contributions, and ensure you have specifically and accurately indicated the role(s) that these authors had in your study. You can update author roles in the Author Contributions section of the online submission form. Please also include the following statement within your amended Funding Statement.*

*“The funder provided support in the form of salaries for authors [insert relevant initials], but did not have any additional role in the study design, data collection and analysis, decision to publish, or preparation of the manuscript. The specific roles of these authors are articulated in the ‘author contributions’ section.” If your commercial affiliation did play a role in your study, please state and explain this role within your updated Funding Statement.*

**RESPONSE:**

As noted above, The funding organization (Lion Corporation) did not play any role in the study design, data collection and analysis, decision to publish, or preparation of the manuscript. Therefore, we have also included the above information in the original Funding Statement and the cover letter. This work was fully funded by Lion Corporation (https://www.lion.co.jp/en/). K Oka, S Uemura, S Morishita, Y Yamamoto, K Kurita are employees of Lion Corporation. Lion Corporation provided support in the form of salaries for authors. We also reviewed the Author Contributions section of the online submission form and confirm that the role(s) that the authors had in this study have been detailed accurately. Therefore, we added to the following sentence to the Funding Statement:

“The funder provided support in the form of salaries for authors [K Oka, S Uemura, S Morishita, Y Yamamoto, K Kurita], but did not have any additional role in the study design, data collection and analysis, decision to publish, or preparation of the manuscript. The specific roles of these authors are articulated in the ‘author contributions’ section.”

*b. Please also provide an updated Competing Interests Statement declaring this commercial affiliation along with any other relevant declarations relating to employment, consultancy, patents, products in development, or marketed products, etc.*

*Within your Competing Interests Statement, please confirm that this commercial affiliation does not alter your adherence to all PLOS ONE policies on sharing data and materials by including the following statement: "This does not alter our adherence to PLOS ONE policies on sharing data and materials.” (as detailed online in our guide for authors http://journals.plos.org/plosone/s/competing-interests) . If this adherence statement is not accurate and there are restrictions on sharing of data and/or materials, please state these. Please note that we cannot proceed with consideration of your article until this information has been declared. Please include both an updated Funding Statement and Competing Interests Statement in your cover letter. We will change the online submission form on your behalf.*

**RESPONSE:**

There are no changes in the Competing Interests Statement. Declarations related to employment have already been described in the original text. As of today (May 16, 2025), the prediction algorithm built in this study is not marketed as a product of Lion Corporation and there is no licensing status for the algorithm. This information will be added to the Competing Interests Statement as needed. In Competing Interests Statement section, I confirmed that this commercial affiliation does not alter our adherence to all PLOS ONE policies, and we added to the following sentence to the Competing Interests Statement section: "This does not alter our adherence to PLOS ONE policies on sharing data and materials.” We have also included information in our cover letter regarding our most recent Funding Statement and Competing Interests Statement.

*6. We note that you have indicated that there are restrictions to data sharing for this study. PLOS only allows data to be available upon request if there are legal or ethical restrictions on sharing data publicly. For more information on unacceptable data access restrictions, please see* [*http://journals.plos.org/plosone/s/data-availability#loc-unacceptable-data-access-restrictions*](http://journals.plos.org/plosone/s/data-availability#loc-unacceptable-data-access-restrictions)*. Before we proceed with your manuscript, please address the following prompts:*

*a) If there are ethical or legal restrictions on sharing a de-identified data set, please explain them in detail (e.g., data contain potentially identifying or sensitive patient information, data are owned by a third-party organization, etc.) and who has imposed them (e.g., a Research Ethics Committee or Institutional Review Board, etc.). Please also provide contact information for a data access committee, ethics committee, or other institutional body to which data requests may be sent.*

*b) If there are no restrictions, please upload the minimal anonymized data set necessary to replicate your study findings to a stable, public repository and provide us with the relevant URLs, DOIs, or accession numbers. For a list of recommended repositories, please see https://journals.plos.org/plosone/s/recommended-repositories. You also have the option of uploading the data as Supporting Information files, but we would recommend depositing data directly to a data repository if possible.*

**RESPONSE:**

Data cannot be shared publicly because of a lack of such description in the study protocol. For items not listed in the research protocol, data sharing is restricted by Instituitonal Review Board of LION Corporation. Data are available from the Instituitonal Review Board of LION Corporation for researchers who meet the criteria for access to confidential data. (Contact via Kazuo Mukasa (Instituitonal Review Board member of LION Corporation), E-mail: mukkaz@lion.co.jp.)

*While revising your submission, please upload your figure files to the Preflight Analysis and Conversion Engine (PACE) digital diagnostic tool,* [*https://pacev2.apexcovantage.com/*](https://track.editorialmanager.com/CL0/https:%2F%2Fpacev2.apexcovantage.com%2F/1/010f01955add63ed-aee6f054-3f4c-4e0d-a73c-31e3d202f3a9-000000/NXNDwY4Gf0_h28NRsOmmerx9egEMat-e9a1J4I38p_A=200)*. PACE helps ensure that figures meet PLOS requirements. To use PACE, you must first register as a user. Registration is free. Then, login and navigate to the UPLOAD tab, where you will find detailed instructions on how to use the tool. If you encounter any issues or have any questions when using PACE, please email PLOS at* [*figures@plos.org*](mailto:figures@plos.org)*. Please note that Supporting Information files do not need this step.*

**RESPONSE:** Following the instructions, we have confirmed using PACE that the figures meet the requirements of PLOS One.

**Response to Reviewer #1:**

*The study addresses a significant public health issue (malocclusion) and explores an innovative AI-based approach for its early detection. Below are some points that need to be improved and clarified in the manuscript.*

**RESPONSE:**

We would like to express our gratitude to you for their insightful comments and suggestions that have helped us improve our manuscript considerably. Please find our responses to the comments below.

***Abstract***

*• Clearly specify the type of study in the methods section.*

*• Indicate where the sample was drawn from.*

*• When stating that questionnaire data were collected, it is important to describe which specific data were included in the algorithm.*

**RESPONSE:**

We have added your suggestions to the Abstract section. The revisions are as follows.

P2, line 11:

This study was conducted as a feasibility study using cross-sectional data.

P2, lines 11–13:

Subjects are recruited from panelists registered with Macromill, Inc. (approximately 1.3 million registered in 2021).

P3, lines 4–6:

Three dental images were used for all model building, and questionnaire data used all four questions (Q1–Q4) for the overbite classification model, Q1 and Q3 for the overjet classification model, and Q1 and Q4 for the crowding classification model.

P13, line 1:

A question number was assigned to each question listed in Table 1.

***Introduction***

*The introduction presents an interesting and relevant study topic but requires refinement in terms of epidemiological accuracy, research justification, and methodological clarity.*

*• While the introduction outlines the prevalence and consequences of malocclusion, it does not explicitly state the core research problem.*

*• Explain the limitations of current detection methods and introduce AI-based image analysis as a potential solution.*

**RESPONSE:**

Thank you very much for your valuable advice. As you suggested, we have revised the explanation of the research issues and limitations of the current detection method and the use of AI-based image analysis as a solution as follows.

∙ P6, lines 6–15

While patients visiting a dentist's office are diagnosed with malocclusion through a detailed examination such as head x-rays, in the field of health checkups and epidemiological studies, such as Dental Aesthetic Index (DAI) and Index of Orthodontic Treatment Need (IOTN) are used to detect malocclusion because precise examinations cannot be performed in the site. These are useful screening tools that quantify the need for orthodontic treatment by incorporating function, morphology, and dentition aesthetics into the evaluation criteria, but they are composed of many items and require a large amount of time for evaluation. Also, it has been reported that the perception of malocclusion varies among evaluators and patients themselves [4]. Therefore, there is a need for a screening tool for malocclusion that is easier to use and has less inter-rater error.

∙ P6, lines 18 – P7, lines 12

Furthermore, Ha *et al*. applied the algorithm of the CNN (convolutional neural network), which is often used in object detection, to dental panoramic images, and they reported that an object detection CNN model based on YOLOv3 was able to detect the presence of a single impacted mesiodens on panoramic radiographs of primary, mixed, and permanent [9]. ~~The expectation is that AI models will be developed for easy assessment of oral health conditions, and patients can receive appropriate feedback regardless of their location.~~

Based on these reports, it is anticipated that algorithms can be developed to enable simple evaluation of oral health by maximizing the use of oral images and other machine learning methods such as CNN, thereby allowing patients to receive appropriate feedback regardless of location. Furthermore, by utilizing these algorithms as screening tools, it is expected that patients' awareness of visiting clinics will increase, enabling screening for oral diseases even in places where detailed examinations are difficult, such as health checkup venues or home.

*• The statement: “The worldwide prevalence of malocclusion is high (up to 54%)” lacks specificity. Prevalence varies significantly based on age group, ethnicity, and classification criteria (e.g., Angle’s classification vs. other methods). The introduction should: specify the population from which this prevalence estimate originates; differentiate between malocclusion severity levels (mild, moderate, severe); and ensure all epidemiological claims are backed by references from peer-reviewed studies.*

**RESPONSE:**

As you indicated, the definition of the population and classification criteria for malocclusion were inappropriate and we has been corrected as follows. Furthermore, since we were unable to confirm details regarding part of the target population extracted from the previously referenced reference No. 1, we have replaced it with another reference. In this reference, the population information and classification criteria for malocclusion were added and the prevalence of malocclusion was described. The revisions are as follows.

∙ P4, lines 2 – 12

~~The worldwide prevalence of malocclusion is high (up to 54%), and its incidence rate is high from primary to permanent dentition [1]~~

The prevalence of malocclusion is high worldwide, and its incidence rate remains at a high level from the deciduous dentition stage to the permanent dentition stage. Alhammadi et al. conducted a systematic review of 53 studies published by November 2016 that met the evaluation criteria and calculated the prevalence of malocclusion [1]. According to the study, during the mixed dentition stage, the average prevalence of malocclusion in Mongoloids was 66.75% for Class I, 22.1% for Class II, and 10.95% for Class III. In Caucasians, the average prevalence was 70.39% for Class I, 25.91% for Class II, and 3.53% for Class III. The results of the above studies indicate that the prevalence of malocclusion in classes I and II is quite high worldwide.

*• The claim that “patients themselves are unaware that the degree of malocclusion is severe enough to require treatment” lacks supporting evidence. The introduction should provide references or data to support this assertion rather than assuming a singular cause.*

**RESPONSE:**

We have added references to support this assertion and made the following modifications.

∙ P5, lines 1–15

Castellote et al. found that the perception of malocclusion varies between individuals and between patients and practitioners [4]. Using frontal intraoral photographs of 24 cases classified by the DAI index, they examined the perception of dental aesthetics among dentists, orthodontists, and the general population. The study showed that there were significant differences in IOTN-AC scores between orthodontists, general dentists, and the general population, with orthodontists giving the most severe scores. This study also suggests that there may be a discrepancy between orthodontists and patients in their perception of the need for orthodontic treatment. In addition, Fujimura et al. compared the students' own perception of dental aesthetics and the results of the dentists' assessment using the IOTN-AC scale in 173 Japanese university students [5]. A comparison between the two showed a large discrepancy. Of the 33 students judged to need orthodontic treatment by the dentists, only 3 (9.1%) were judged to need orthodontic treatment by the students' self-assessment. These studies suggest that some patients themselves may not be aware that the degree of malocclusion is severe enough to require treatment.

*• Clearly define the acronym OECD* *(Organization for Economic Cooperation and Development) when it first appears in the introduction.*

**RESPONSE:**

Thank you for pointing this out. We have defined the acronym OECD.

∙ P5, lines 16–17

A previous study of nine OECD (Organization for Economic Cooperation and Development) member countries with low DMFT (Decayed, Missing, and Filled Teeth) indices found that Japan's regular dental visit rate at age 12 was 44%,

*• The introduction references AI-based classification but does not outline the type of AI model being considered (e.g., CNNs, deep learning, transfer learning) and the challenges in training AI for malocclusion detection (e.g., dataset bias, image quality issues, need for expert annotations). A brief mention of these challenges would help frame the study’s contribution in a realistic context.*

**RESPONSE:** We have outlined the issues involved in constructing an AI model for malocclusion, and have emphasized the position of this research.

∙ P6, lines 18 – P7, lines 4

Furthermore, Ha *et al*. applied the algorithm of the CNN (convolutional neural network), which is often used in object detection, to dental panoramic images, and they reported that an object detection CNN model based on YOLOv3 was able to detect the presence of a single impacted mesiodens on panoramic radiographs of primary, mixed, and permanent [9].

∙ P7, lines 4- P8, lines 8

~~The expectation is that AI models will be developed for easy assessment of oral health conditions, and patients can receive appropriate feedback regardless of their location.~~

Based on these reports, it is anticipated that algorithms can be developed to enable simple evaluation of oral health by maximizing the use of oral images and other machine learning methods such as CNN, thereby allowing patients to receive appropriate feedback regardless of location. Furthermore, by utilizing these algorithms as screening tools, it is expected that patients' awareness of visiting clinics will increase, enabling screening for oral diseases even in places where detailed examinations are difficult, such as health checkup venues or home.

However, in addition to the fact that there is no reports on AI models for determining malocclusion during the mixed dentition period, in order to make the prediction algorithm available to the general public at home or health checkup venues, it is necessary to construct an algorithm using dental images that can be taken at home, rather than panoramic photographs taken at dental clinics. Furthermore, intraoral photographs taken at dental clinics are often taken for the purpose of diagnosing malocclusion, which may result in a biased dataset with a high proportion of individuals with malocclusion. To build an AI model that detects malocclusion, it is necessary to also collect images that do not show malocclusion abnormalities, and data diversity is required.

~~However, no study has reported the development of AI models for judging the state of malocclusion in children. Furthermore, no study has been conducted on AI models for assessing oral conditions using data that can be collected at home, such as images captured using a smartphone camera.~~

***Methods***

*• The study is described as a pilot study, yet it includes 520 participants. The authors should clarify if this is truly a pilot study or an exploratory AI development study. If it is a pilot study, explain why such a large sample was used, and If it is a full-scale study, provide justification for the sample size based on power calculations.*

**RESPONSE:**

Thank you for pointing this out. As this research is an exploratory AI development study, we have changed the notation from “pilot study” to “feasibility study”.

P8, lines 18 – P9, lines 2:

~~This was a~~ ~~pilot study including elementary school children (grades 3–6)~~.

This study was conducted as a feasibility study using cross-sectional data and included elementary school children (grades 3–6).

*•The recruitment method via Macromill Inc. (a commercial survey company) may introduce selection bias: Were participants from diverse socioeconomic backgrounds? Were they representative of the general population of Japanese children? How was participant diversity ensured?*

**RESPONSE:**

Macromill has a panel of approximately 1.3 million people (the number of registered panelists at of the time of this research survey in 2021), and it is a diverse group. The panelists reside in various areas ranging from Hokkaido to Kyushu. The following is information on the panelists' place of residence: Hokkaido area 4.36%, Tohoku area 5.57%, Kanto area 38.35%, Chubu area 16.75%, Kinki area: 17.98%, Chugoku area:5.10% Shikoku area:2.32% Kyusyu area:9.58%. In addition, the household income of Macromill panelists range widely, from less than 2 million yen to more than 20 million yen. The ratio of household income for Macromill panelists is as follows (less than 2 million yen: 10.5%, 2-4 million yen: 25.7%, 4-6 million yen: 26.9%, 6-8 million yen: 17.5%, 8-10 million yen: 9.9%, 10-12 million yen : 4.9%, 12 million yen to less than 15 million yen: 2.5%, 15 million yen to less than 20 million yen: 1.2%, 20 million yen or more: 0.8% (excluding those who did not respond). The most common household income among Macromill panelists was between 4 million and 6 million yen. In a report on a Comprehensive Survey of Living Conditions by the Japanese Ministry of Health, Labor and Welfare (<https://www.mhlw.go.jp/english/database/db-hss/cslc-report2022.html>), the average household income as of 2021 was 5.457 million yen, and the median was 4.230 million yen, suggesting that the economic background of the Macromill panelists is not very different from that of the general population. We have added the background information on Macromill panelists.

P9, lines 13-17

Participants in this study were recruited from among the 1.3 million panelists members registered with Macromill, Inc. as test participants at the time of the 2021 survey (Macromill's registered panelists represent a diverse group in terms of residence and household income that does not differ greatly from the general population).

*• Describe how the sampling method ensures generalizability and address potential selection bias.*

**Response:**

Information about the test was sent to all registered panelists, and registration was closed on a first-come, first-served basis. For this reason, we did not make any adjustments, such as setting a maximum number of participants per prefecture. However, as mention aboved, because the panelist population itself is fairly diverse, we assumed that we were able to ensure a certain degree of generality. The following shows the residential location information and household income status of the 519 children who participated in this study. As shown below, we have assumed that the population of this study is not very different from the general population in terms of residential background and household income.

・Residenial location (of 519 participants):

Hokkaido area:4.43%, Tohoku area:4.82%, Kanto area:32.18%, Chubu area:19.08%, Kinki area: 20.04%, Chugoku area:5.97%, Shikoku area:2.89%, Kyusyu area:10.60%. The percentages are not much different from the demographic breakdown of the Japanese population.

・Annual household income(of 519 participants):

under 2 million yen: 10.5%, 2 to under 4 million yen: 25.7%, 4 to under 6 million yen: 26.9%, 6 to under 8 million yen: 17.5%, 8 to under 10 million yen: 9.9%, 10 to under 12 million yen : 4.9%, 12 to under 15 million yen: 2.5%, 15 to under 20 million yen: 1.2%, 20 or more million yen: 0.8%, no answer: 13.9%. As mentioned previously, in a report on a Comprehensive Survey of Living Conditions showed that the average and median household income was between 4 and 6 million yen, and the most common household income among children who participated in the test was between 4 and 6 million yen. It suggested that the economic background of the children who participated in the test is not much different from that of the general population. The background information for the above subjects is given in the method section. The corrections are as follows.

P21, lines 18 – P22, lines 11

The following is a breakdown of the residential areas and annual household incomes of the 519 children recruited this time.

Residential area: Hokkaido area 4.43%, Tohoku area 4.82%, Kanto area 32.2%, Chubu area 19.1%, Kinki area: 20.0%, Chugoku area:5.97%, Shikoku area:2.89%, Kyusyu area:10.6%.

Annual household income: under than 2 million yen: 10.5%, 2 to under 4 million yen: 25.7%, 4 to under 6 million yen: 26.9%, 6 to under 8 million yen: 17.5%, 8 to under 10 million yen: 9.9%, 10 to under 12 million yen : 4.9%, 12 to under 15 million yen: 2.5%, 15 to under 20 million yen: 1.2%, 20 or more million yen: 0.8%, no answer: 13.9%. The above percentages for annual household incomes show no much difference from the figures from the national census conducted by Japanese government agency for the entire country [23].

*• It would be beneficial to add a section detailing quality control measures for smartphone images and any inter-rater agreement measures.*

**RESPONSE:**

Thank you for your advice. We have added the following information regarding the above. Firstly, to ensure image quality, we excluded participants who had smartphones with outdated operating systems and only included participants who had smartphones with Android 6.0 or higher or iOS 11.0 or higher. We added this information about participant requirements to the Methods section. In addition, as a measure to ensure image quality, we presented detailed photographic conditions on the smartphone app interface screen before taking the photo. We also added this information about participant requirements to the Methods section. The detailed method is as follows.

P9, lines 5–7

Furthermore, this study included participants who had smartphones with Android 6.0 or higher or iOS 11.0 or higher.

P11, lines 16 – P12, lines 5

In addition, before the shooting guide frame is displayed, the following instructions were displayed on the application screen to standardize the shooting conditions. The application interface is designed to require users to press the Confirm button after reading the instruction. The instructions for shooting conditions are as follows. (a) take the photo in a well-lit place, (b) take the photo with the height of the child's face and the height of the smartphone camera aligned, (c) take the photo with the back teeth firmly clenched, and (d) take the photo with the lips open so that the teeth are visible).

We have also added information related to annotation of malocclusion in the Methods section.

P14, lines 12–16

When annotating the dental images collected, unclear images were excluded. After that, three researchers simultaneously evaluated whether or not each subject had malocclusion. Before annotation, sample images of malocclusion were reviewed, and the three researchers calibrated their judgments before beginning annotation.

*• What specific machine learning (ML) algorithms were used? (e.g., CNNs, decision trees, SVM, etc.)*

**RESPONSE:**

We have added more details about the algorithm used in the Methods section. The algorithm used in the classification model for malocclusion we have constructed is as follows.

P17, lines 3 – P18, lines 2:

The following modeling was performed by Datarobot on autopilot. Questionnaire data and image data were automatically separated from the dataset, and feature extraction from the image data was performed by a pre-trained deep learning model of SqueezeNet, a type of CNN that had been pre-trained. Next, Datarobot used the binary data from questionnaire data and the extracted image features as explanatory variables to create a classification model for the presence or absence of overbite using the Elastic Net Classifier.

In the overjet classification model, before building the model using all data sets, Datatobot only used the feature values extracted by SqueezeNet, and calculated the predicted value for the presence or absence of overjet using the Elastic Net Classifier. Then, Datarobot used the predicted value itself as a new feature value, integrated it with the binary data from the questionnaire data, and constructed an algorithm for judging overjet using the Elastic Net Classifier.

In the crowding classification model, before building the model using all data sets, Datarobot used only the image features extracted by SqueezeNet as explanatory variables and calculated predictions for the presence or absence of crowding using the Elastic Net Classifier. Datatobot adopted the above prediction values themselves as new features. By feeding the binary data from the questionnaire and the prediction values into the SVM Classifier model as explanatory variables, we constructed a crowding classgfcation model.

*• How was model selection performed beyond AutoML automation?*

*• What hyperparameter tuning methods were used?*

**RESPONSE:**

DataRobot is very good at selecting the optimal hyperparameters for a model to maximize accuracy. All model building, including hyperparameter tuning, was done using DataRobot's AutoML feature. In terms of model selection, among the multiple models built, the model with the highest AUC value in five cross-validations was selected as the binary classification model. Note that even if the AUC value is high, models that only focus on other areas such as fingers instead of the dental area in the activation MAP were considered inappropriate models and were not selected. We added information regarding model selection to the Method section.

P17, lines 6 – 10

In terms of model selection, among the multiple models built, the model with the highest AUC value in five cross-validations was selected as the binary classification model. We considered inappropriate models and didn't selected models that only focus on other areas such as fingers instead of the dental area in the activation MAP even if the AUC value is high.

*• The blender models mentioned are ensemble models, but were any baseline models (e.g., logistic regression, traditional orthodontic assessments) used for comparison? How did the chosen models perform against existing AI models in dentistry?*

**RESPONSE:**

Since there are no previous reports on the use of AI algorithms for malocclusion in school-age children, a direct comparison is considered difficult. However, for comparison purposes, we have constructed a model using binary logistic regression analysis based on questionnaire data alone to determine the presence or absence of malocclusion, which was performed on Datarobot. We created a new table summarizing the performance results of the logistic regression analysis model and presented it in Table 3. Logistic regression is commonly used as a screening tool in the medical field, but as shown in Table 3, logistic regression models do not perform adequately, and it is believed that machine learning models using dental images are more useful for determining malocclusion. In addition, we have revised the Methods and Results section to reflect the addition of the table as follows.

P20, lines 10 – P21, lines 2

**Comparison with logistics regression model**

In order to compare the performance of the constructed machine learning algorithm with the classic logistic regression model, we have constructed a model using binary logistic regression analysis based on questionnaire data alone to determine the presence or absence of malocclusion, which was performed on Datarobot. Note that all four questionnaire data sets shown in Table 1 were used as explanatory variables to construct these logistic regression models. As with the method described above, we divided the entire dataset into two random groups to build a logistic regression model. We used 80% of the data as a training dataset for model construction and the remaining 20% as a validation dataset for new data. We then used the validation dataset to calculate the performance metrics described above and evaluate the model's performance.

P26, lines 8 – P27, lines 5

**Performance metrics for logistic regressions models on new data**

The three logistic regression models showed low performance with an AUC of less than 0.70 (Table 3). The overbite model showed low performance, with an AUC of 0.52, sensitivity of 100.0%, specificity of 0.0%, accuracy of 36.6%, precision of 36.6%, and F1 score of 0.5354. The overjet model also showed low accuracy, with an AUC of 0.55, sensitivity of 100.0%, specificity of 4.1%, and accuracy of 24.7%, precision of 22.2%, and F1 score of 0.3636. The crowding model also showed low accuracy, with an AUC of 0.62, sensitivity of 48.0%, specificity of 75.3%, and accuracy of 68.4%, precision of 40.0%, and F1 score of 0.4364.

| **Class** | **Sensitivity**  **(%)** | **Specificity**  **(%)** | **Accuracy**  **(%)** | **Precision**  **(%)** | **F1 score** | **AUC** |
| --- | --- | --- | --- | --- | --- | --- |
| **Overbite** | 100.0 | 0.0 | 36.6 | 36.6 | 0.5354 | 0.52 |
| **Overjet** | 100.0 | 4.1 | 24.7 | 22.2 | 0.3636 | 0.55 |
| **Crowding** | 48.0 | 75.3 | 68.4 | 40.0 | 0.4364 | 0.62 |

**Table 3. Performance metrics of the logistic regression models on the test dataset.**

AUC: area under the receiver operating characteristic curve.

P35, lines 3–8

For comparison, a binary classification model was also created using logistic regression with only the questionnaire data. The results showed that the logistic regression model had poor classification accuracy for all types of malocclusion (AUC < 0.70), suggesting that questionnaire data alone may not be sufficient for accurately classifying malocclusion. Based on these results, it was suggested that machine learning models using dental images are useful for constructing an algorithm for classifying malocclusion.

*• The study uses AUC-ROC, sensitivity, specificity, and accuracy, but the precision and F1-score are missing, and these are crucial for imbalanced datasets.*

**RESPONSE:** Thank you for your insgihtful comments. Based on your advice, I have added the data to Table 2. I have also added these indicators in abstract, method and discussion section.

P2, lines 18 – P3, lines 2

Using a test dataset, the model’s performance was assessed through sensitivity, specificity, accuracy, precision, F1 score, receiver operating characteristic (ROC) curves, and area under the ROC curves (AUC).

P19, lines13 – 15

The performance of the constructed models was assessed in terms of sensitivity, specificity, accuracy, precision, and F1 score ~~and accuracy~~.

P20, lines 4 – 5

Precision = TP / (TP + FP)

F1 score = 2(Precision×Sensitivity) / (Precision + Sensitivity)

P25, lines 5 – P25, lines 6

**Performance metrics for** **machine learning models on new data**

~~The three models scored high values for each performance metrics (Table 2). The overbite model maintained high performance in detecting overbite, with an AUC of 0.92, sensitivity of 91.2%, specificity of 81.4%, and accuracy of 82.3%. The overjet and crowding classification models showed moderate accuracy, with an AUC of 0.74, sensitivity of 70.0%, specificity of 71.2%, and accuracy of 71.0% and AUC of 0.73, sensitivity of 80.0%, specificity of 61.6%, and accuracy of 66.3%, respectively.~~

The three machine learning models showed high performance, with an AUC of 0.70 or higher, which is an overall accuracy metric (Table 2). The overbite model maintained high performance in detecting overbite, with an AUC of 0.92, sensitivity of 91.2%, specificity of 81.4%, accuracy of 85.0%, precision of 73.8%, and F1 score of 0.8158. The overjet model showed moderate accuracy, with an AUC of 0.74, sensitivity of 70.0%, specificity of 71.2%, and accuracy of 71.0%, precision of 40.0%, and F1 score of 0.5091. The crowding model also showed moderate accuracy, with an AUC of 0.73, sensitivity of 80.0%, specificity of 61.6%, and accuracy of 66.3%, precision of 41.7%, and F1 score of 0.5479.

| **Class** | **Sensitivity**  **(%)** | **Specificity**  **(%)** | **Accuracy**  **(%)** | **Precision**  **(%)** | **F1 score** | **AUC** |
| --- | --- | --- | --- | --- | --- | --- |
| **Overbite** | 91.2 | 81.4 | 85.0 | 73.8 | 0.8158 | 0.92 |
| **Overjet** | 70.0 | 71.2 | 71.0 | 40.0 | 0.5091 | 0.74 |
| **Crowding** | 80.0 | 61.6 | 66.3 | 41.7 | 0.5479 | 0.73 |

**Table 2. Performance metrics of the machine learning models on the test dataset.**

AUC: area under the receiver operating characteristic curve.

P34, lines 13 – P35, lines

All models showed high performance in recall value, but the precision value were poor for the overjet and crowding models. This indicates that although the constructed models have a low false negative rate, they tend to produce a certain number of false positives. Therefore, the current model is not suitable for precise diagnosis, but we think that it is suitable as a screening tool in environments where precise diagnosis is difficult (e.g., health check-ups). In the future, it is suggested that further improvements to the model are necessary to implement it as a screening tool while maintaining a high recall value and improving the precision value.

*• In the statistical analysis, was any adjustment made for potential confounding factors?*

**RESPONSE:**Thank you for your comments. In this study, adjustment for confounding factors was not performed in statistical analysis for the purpose of exploratory analysis. The above content is described as a limitation of the study in the Discussion section.

P35, lines13 – 18

In this study, we selected specific oral habits that have been suggested to be associated with malocclusion and constructed classification models as exploratory analysis. In the future, we will need to comprehensively collect and analyze questionnaire data while controlling for confounding factors. This will allow us to narrow down the factors that are more strongly associated with specific malocclusions and refine the algorithm.

**Results**

*• In the results tables, I suggest including p-values instead of n.s. (not significant).*

**Response:**Thank you for your advice. In the Table 4–6 (P27–P28), we have revised the notation to include the p-value instead of n.s..

***Discussion***

*• The discussion does not mention whether the model's performance holds up under real-world conditions or external datasets.*

*• It does not acknowledge how image distortions, lighting conditions, and smartphone differences may have affected model accuracy.*

**Response:** Thank you for your insightful comments. Based on your comments, we have added the following to the limitations of the study and future challenges.

P37, lines 12 – 13

Under real-world conditions, the angles of dental images taken may become even more diverse.

P37, lines 15 – P38, lines 13

Furthermore, to minimize the impact of the angle of view of the captured images, improvements to the application screen instructions and the UI design of the image capture screen are also considered necessary for the implementation of this algorithm. Third, in addition to the angle of view, it will be necessary to verify the versatility of the system in relation to differences in smartphone models and shooting environments. In this study, although we limited our analysis to participants who own smartphones running Android 6.0 or later or iOS 11.0 or later, and we excluded images that were too dark from the analysis, we did not restrict the brightness (Lux) of the lighting or the smartphone models. Therefore, we believe that we were able to collect images under a certain degree of diverse conditions. The AUC for three classification model exceeded 0.70 in 5-fold cross-validation and accuracy verification using a test dataset, suggesting that the model demonstrates consistent accuracy across diverse environments. However, it is necessary to verify whether similar results can be obtained with images taken using a wider range of smartphone models or older versions of smartphones. Furthermore, when implementing this model in an application, it is also important to implement logic to select input images, such as excluding overly dark images based on color values (e.g., RGB).

*• Address the need for external validation using independent datasets*

**Response:**

Thank you for your comments. we have mentioned the need for external validation as following text.

P39, lines 5 – 8

Furthermore, external validation using an independent dataset is necessary. Leveraging the advantages of the application, we plan to collect images on a larger scale through the application and consider accuracy validation using multiple external datasets beyond the current trial in the future.

*• The discussion only focuses on sensitivity and accuracy but does not discuss misclassification cases. Include an error analysis, discussing false positive and false negative rates and explain the potential clinical consequences of these misclassifications.*

**Response:**

Thank you for your valuable comments. As mentioned the above, we have included an explanation of misclassification in the Discussion section. We consider that this algorithm is useful as a screening tool in situations where precise diagnosis is not possible, such as health checkups.

*•The discussion claims that questionnaire data had little impact on overbite predictions but was more relevant for overjet and crowding. However, it does not explain why this variation occurs.*

*• The relationship between oral habits and malocclusion should be discussed in greater depth, particularly how AI can or cannot capture behavioral influences. Provide a deeper theoretical explanation of why oral habits impact certain malocclusions more than others.*

**Response:**

Thank you for your insightful comments. First, while there are reports that suggest an association between genetic factors and overbite, there are no reports that clearly demonstrate an association between specific oral habits and overbite. In contrast, the oral habits used as factors in the construction of the algorithm in this study (such as keeping the mouth open and chewing habits) have been shown to be associated with overjet or crowding. In the Discussion section, we have revised the explanation of why these oral habits contribute to the development of certain malocclusions.

P31, lines 11 – P32, lines 5

Children with normal breathing patterns form a sealed oral cavity with the lips closed and the tongue in contact with the palate and lingual side of the upper dentition. Balanced muscle strength between the internal muscles of the tongue and the external muscles of the lips and cheeks is essential for normal development of the maxillary arch. In contrast, keeping the mouth open may result in mouth breathing with the tongue positioned forward or downward, lowering the mandible and potentially causing muscle imbalance. It may result in not only a lack of expansion stimulus to the maxillary side caused by the tongue contact, but also the promotion labial inclination of the maxillary incisors by lack of oral closure, potentially leading to oral cavity and craniofacial anomalies. A cross-sectional study has shown that mouth breathers have significantly more lip closure insufficiency compared to nose breathers, and mouth breathers have significant backward and downward rotation of the mandible and increased overjet compared to nose breathers [24].

P32, lines 17 – P33, lines 2

Crowding is a malocclusion caused by insufficient space for teeth to align properly. It has been suggested that immature jaw development due to the habit of chewing soft foods may contribute to aggravation of crowding.

P33, lines 7 – 13

In particular, the median palatine suture is a growth site in maxillary expansion and plays a crucial role in the lateral growth of the maxilla. High masticatory loads are suggested to contribute to maxillary growth through stimulation of these sutures. The above animal experiments have reported that in group where loads were reduced by a soft diet, the width of the palatine sutures decreased compared to group fed a hard diet, and returning to a hard diet induced reactivation of the suture region.

P35, lines 9 – P36, lines 7

Furthermore, the results of permutation importance results showed that not only dental images but also the presence or absence of these oral habits related to open-mouth posture and chewing habits are important in determining of crowding and overjet.

However, malocclusion is a multifactorial disease, and other oral habits may also contribute to the development of malocclusion. In this study, we selected specific oral habits that have been suggested to be associated with malocclusion and conducted exploratory analyses. In the future, it will be necessary to comprehensively collect questionnaire data, analyze them while controlling for confounding factors, and narrow down the factors that are thought to be more strongly associated with specific malocclusions in order to refine the algorithm. However, It should be noted that information on oral habits to be included in the algorithm should be limited to those that can be identified by parents and general public, such as open mouth posture or chewing habits. Considering the application and convenience of the model, it is necessary to limit the explanatory variables input into the algorithm to items that can be easily determined. Some oral habits, such as tongue thrust swallowing, can only be determined by a specialist dentist. Going forward, we plan to further refine the questionnaire data while considering the limitations of oral habit information that can be input into this algorithm.

*• Address potential dataset biases and the need for diverse, representative training data and discuss ethical concerns regarding AI-based self-diagnosis and whether these models should only be used as a pre-screening tool.*

**Response:**

Thank you for your valuable comments, As mentioned above, we added the need for diverse training data in the Discussion part. The algorithm developed in this study was not intended for precise diagnosis, but rather as a screening tool to encourage dental visits. As you pointed out, these algorithm may pose a risk of users being satisfied with self-diagnosis and missing opportunities for dental visits. To prevent the loss of dental visits after self-diagnosis, it is essential to design a user interface that encourages dental visits after screening and provide information on oral health to promote behavioral change. Therefore, in addition to improving the accuracy of the algorithm, it is necessary to design specifications that address ethical concerns. To clarify the position of this algorithm, we have added the following text.

P39, lines 8 – 15

Finally, it is necessary to confirm whether the early detection of malocclusion using this algorithm can promote changes in patients' health-seeking behavior. In addition, to prevent missed dental visits after self-diagnosis, it is necessary to design a user interface that encourages dental visits after screening and to implement informational screens that promote behavior change. In summary, in addition to improving the accuracy of these algorithms, it is important to consider the design specifications for a screening tool that can effectively promote behavior change in patients in the future.

**Response to Reviewer #2:**

*This study presents an innovative approach to diagnosing malocclusions in children using artificial intelligence (AI) based on photographs taken by their caregivers. The topic is highly relevant, particularly given the expanding role of AI in Dentistry and the feasibility of remote methods for screening and monitoring.*

**RESPONSE:**

We sincerely appreciate your insightful comments and suggestions, which have helped us to significantly improve our manuscript. Our responses to the comments are provided below.

*However, a critical methodological limitation must be addressed: the lack of clinical validation of the AI model by experienced orthodontists. Without this comparison, it is not possible to determine the system’s accuracy or practical applicability. Since orthodontic diagnosis is inherently complex, validation against human experts is essential to ensure that the model correctly identifies malocclusion patterns. I recommend that the authors consider the following approaches to strengthen the study:*

Include a preliminary validation – If feasible, the authors could compare the AI-generated diagnoses with those made by orthodontists in a selected sample.

**RESPONSE:**

Thank you fou your insightful comments. As you pointed out, the algorithm developed in this study was not directly compared with diagnostic values obtained by orthodontists. Therefore, verification of its clinical applicability remains a future research task. However, in the protocol of the research plan conducted this time, there is no mention of performing diagnostic comparisons with orthodontists for the developed algorithm, and the participants' consent for such comparisons was not obtained. Therefore, it is difficult to implement at this time. In the future, we plan to conduct a new study based on the developed algorithm and obtain consent from participants to verify its accuracy as the next study. We have added the above limitations of the study and the need for further investigation as future research in the discussion section.

P38, lines 13 – P39, lines 5

Fourth, to verify the accuracy of the algorithm, it is necessary to compare the prediction results of the developed algorithm with the diagnostic values obtained by several orthodontists. In this study, we have not yet verified the extent to which the prediction values of the constructed algorithm agree with the diagnostic results of experienced orthodontists. In future research, it is important to compare the predictive accuracy of AI with the diagnostic accuracy of experienced orthodontists, construct a more reliable AI model, and further verify its clinical practicality.

~~Third, the accuracy of the algorithm should be verified by comparing the predictions of the developed algorithm with diagnostic values obtained from several orthodontists. Future studies should compare the accuracy of AI predictions with that of diagnoses made by skilled orthodontists to build more reliable AI models.~~

*Additionally, it would be helpful for the authors to clarify: How the images were standardized to minimize variations (e.g., lighting, capture angle, resolution).*

**RESPONSE:**

Thank you for your comments. To minimize variations in image brightness and angle, image capture instructions were displayed on the application screen prior to image capture. In addition, participants with smartphones running older operating systems were excluded to control image quality. The above image quality control methods have been added to the Method section below.

P9, lines 5 – 7

Furthermore, this study included participants who had smartphones with Android 6.0 or higher or iOS 11.0 or higher.

P11, lines 16 – P12, lines 5

In addition, before the shooting guide frame is displayed, the following instructions were displayed on the application screen to standardize the shooting conditions. The application interface is designed to require users to press the confirm button after reading the instruction. The instructions for shooting conditions are as follows. (a) take the photo in a well-lit place, (b) take the photo with the height of the child's face and the height of the smartphone camera aligned, (c) take the photo with the back teeth firmly clenched, and (d) take the photo with the lips open so that the teeth are visible).

**Response to Reviewer #3:**

*I congratulate you on the idea of the article for being original and for the new insights, but some methodological issues regarding the study design and methodology need to be made clearer in the study.*

**Response:**

We sincerely appreciate the insightful your comments, we were able to make significant improvements to the manuscript. Our responses to the comments are as follows.

*First, it is important to include a subtopic in the methodology that specifies the study design. From the title, “classification algorithm”, it is possible that this study is cross-sectional. It is important to state in the methodology, perhaps in the description of the analysis, that The structure of the present manuscript followed the guidelines Developing and Reporting Machine Learning Predictive Models in Biomedical Research.*

**Response:**

Thank you very much for your valuable feedback. We have added a note to the method section to clarify that this study is a cross-sectional study. We also appreciate you sharing the guidelines with us. We have confirmed that this paper complies with the above guidelines and have noted this in the paper.

P2, lines 11

This study was conducted as a feasibility study using cross-sectional data.

P8, lines 16 – 17

The structure of the present manuscript followed the guidelines for Developing and Reporting Machine Learning Predictive Models in Biomedical Research [10].

P8, lines18 – P9, lines2

~~This was a pilot study including elementary school children (grades 3–6)~~.

This study was conducted as a feasibility study using cross-sectional data and included elementary school children (grades 3–6).

*Many points are not clear in the analysis. The first point is whether variables with missing data were excluded from the analysis and what strategies were adopted to obtain the inputs for developing the algorithm.*

**Response:**

Thank you for your valuable feedback. First, since there was no mention of how missing values were handled, we have added the following information. Secondly, we have added details about the algorithm development used in this study below.

P12, lines 12 – 16

In addition, the number of dental images collected from the 519 participants is as follows: 519 frontal dental images, 513 right side dental images, and 512 left side dental images, for a total of 1,544 images. There were no missing values in the questionnaire data, and responses were obtained from all 519 participants.

P14, lines 4 – 6

In the variables shown in Table 1, missing information was not supplemented with representative values or other substitute values, and was treated as missing values when constructing the algorithm.

P17, lines 3 – P18, lines 2:

The following modeling was performed by Datarobot on autopilot. Questionnaire data and image data were automatically separated from the dataset, and feature extraction from the image data was performed by a pre-trained deep learning model of SqueezeNet, a type of CNN that had been pre-trained. Next, Datarobot used the binary data from questionnaire data and the extracted image features as explanatory variables to create a classification model for the presence or absence of overbite using the Elastic Net Classifier.

In the overjet classification model, before building the model using all data sets, Datatobot only used the feature values extracted by SqueezeNet, and calculated the predicted value for the presence or absence of overjet using the Elastic Net Classifier. Then, Datarobot used the predicted value itself as a new feature value, integrated it with the binary data from the questionnaire data, and constructed an algorithm for judging overjet using the Elastic Net Classifier.

In the crowding classification model, before building the model using all data sets, Datarobot used only the image features extracted by SqueezeNet as explanatory variables and calculated predictions for the presence or absence of crowding using the Elastic Net Classifier. Datatobot adopted the above prediction values themselves as new features. By feeding the binary data from the questionnaire and the prediction values into the SVM Classifier model as explanatory variables, we constructed a crowding classgfcation model.

*The article does not mention whether other classification models were tested, in addition to SVM. If other algorithms such as random forest, KNN, XGboost were tested, it would be essential to mention the methodology and organize a table with the information found for these tested algorithms. I suggest including, in addition to sensitivity, specificity, AUC, other metrics, such as F-1, accuracy and recall.*

**Response:**

Regarding the verification of other algorithms, we also constructed a model using logistic regression, a classical method, and performed accuracy verification. As mentioned above, we created a new table summarizing the performance results of the logistic regression analysis model and presented it in Table 3(P27, lines 4). Logistic regression is widely used as a screening tool in the medical field, but as shown in Table 3, the logistic regression model did not show sufficient performance in determining malocclusion in children. Therefore, we believe that the machine learning model using dental arch images is useful for determining malocclusion. Finally, regarding the other performance metrics, thank you for your insightful suggestions. As you pointed out, data was missing for the F1 Score and Precision metrics, so we reanalyzed the data and added the numbers to Table 2(P26, lines 5). While the overjet and crowding models showed low precision, all three models showed high performance in terms of recall. Based on the above, although the current models are not suitable for precise diagnosis, they are expected to be useful as screening tools for broad detection of positive cases in settings where precise diagnosis is difficult (e.g., health checkups).

*It would also be interesting to point out, in addition to the associations, the degree of importance of the input variables. This can be represented by a graph modified by SHapley Additive exPlanations.*

*Congratulations on the article! I recommend including this information and that the discussion consider these aspects.*

**Response:**

Thank you very much for your advice. As a result of calculating the SHAP values, the contribution of explanatory variables in each model was as follows. We calculated the mean absolute SHAP value for each explanatory variable and, similar to permutation importance, presented the scores as relative ratios of the other factors when the explanatory variable with the highest SHAP value was set to 100%.

■Relative ratio for SHAP value in overbite classification model

Frontal image:100%

Right image:38.2%

Left image:37.2%

Q3:0.0647%

Q1:0.0510%

Q2:0.0249%

Q4:0.0157%

■Relative ratio for SHAP value for overjet classification model

Frontal image:100%

Right image:78.9%

Left image:27.2%

Q1:16.9%

Q3:6.33 %

■Relative ratio for SHAP value for crowding classification model

Frontal image:100%

Right image:30.0%

Q4:23.1%

Left image:22.9%

Q1:2.36 %

As a result of analyzing the importance of the explanatory variables in each model using SHAP values, it became clear that the results were roughly consistent with the permutation importance analysis results shown in Figure 4–6. In all classification models, frontal images contributed the most to the results. Furthermore, in the overbite classification model, the dental images contributed mainly, while the SHAP values of the questionnaire data were quite low. In addition, in the overjet and crowding classification models, it became clear that the questionnaire data contributed to the classification to some extent, and the results were similar to those obtained from the permutation importance analysis. Therefore, it is thought that the interpretation of the importance of the explanatory variables remains unchanged, we have not revised the figure at this time. However, if it would be better to replace the result of permutation importance with the results of these SHAP scores, we would be happy to revise it. Thank you very much for your valuable feedback.

**To academic editor:**

We have reviewed the manuscript and found some missing references and typographical errors. We have made the following corrections.

First, I added and revised the references used for annotating malocclusion.

P14, lines 10 – 12

The dentition and occlusal status of children were determined from smartphone dental images based on the School Dentist Guidelines of the Japan Association of School Dentists and previous study [13].

P37, lines 6 – 7

This study has several limitations. First, the dentition and occlusal status were determined based on the school dentist’s guidelines and previous study [13].

Second, regarding the explanations for the questions listed in Table 1, question numbers have been added for clarity.

P13, lines 1

**Table 1. Each variable for the dataset.**

Q1: Is your child’s mouth often open during the day?

Q2: Does your child sleep with their mouth open?

Q3: Does your child have difficulty eating hard foods?

Q4: Does your child often prefer soft foods?

Third, to avoid ambiguity, we have divided the “Data Preparation (including data cleansing and annotation)” part(P14, lines 8) described in the Method section into separate chapters on “Data annotation” and “Data cleansing for machine learning,” and described the changes in the data set in each chapter. In this study, statistical analysis was performed using the dataset after data annotation. When constructing the malocclusion detection algorithm, the dataset was further refined through data cleansing after data annotation to create a dataset specifically for algorithm construction, which was then used for analysis. To clarify this progression, we have revised the flow in Figure 1 and the Method section as follows.

P14, lines 8 – P15, lines3

**~~Data preparation (including data cleansing and annotation )~~**

**Data annotation**

The dentition and occlusal status of children were determined from smartphone dental images based on the School Dentist Guidelines of the Japan Association of School Dentists and previous study [13]. When annotating the dental images collected, unclear images were excluded. After that, three researchers simultaneously evaluated whether or not each subject had malocclusion. Binary data on the presence and absence of dental malocclusion (overbite, overjet, and crowding) were obtained from smartphone dental images. Those with no clearly imaged dentition were excluded from the analysis before annotation. The breakdown of each malocclusion data after data annotation is shown below (overbite (n=166) / no overbite (n=342), overjet (n=147) / no overjet (n=368), crowding (n=123) / no crowding (n=395)).

P15, lines 5 – 18

**Data cleansing for machine learning**

To construct a classification model using deep learning, the annotated data were further cleaned. If at least one of the images of the three teeth in the anterior region, left side, or right side of the dentition was not taken at the central occlusion position, or if the anterior teeth of the mandible were not properly captured, the subject was excluded from the dataset used to construct the algorithm. For dental images that were not in the central occlusal position, ~~overbite or overjet was not determined, and~~ the images were not adopted in the training dataset for overbite or overjet classification model ~~this malocclusion~~. Additionally, if the mandibular anterior teeth were not adequately captured due to overbite or other reasons, ~~crowding was not determined, and~~ the images were not adopted in the training dataset for crowding classification model. The breakdown of each malocclusion data after data cleansing for machine learning is shown below(overbite (n=155) / no overbite (n=308), overjet (n=138) / no overjet (n=329), crowding (n=112) / no crowding (n=381)).

Fourth, after reviewing the “MANUSCRIPT BODY FORMATTING GUIDELINES” for PLOS one again, we found that the font size for the level 3 heading for the subsection in the Method section was inappropriate, so we have corrected it to the specified font size (14pt font). Finally, based on your comments, we have changed some references and made corrections as indicated in the Reference section.

Thank you again for giving us the opportunity to improve our manuscript with your insightful comments. We have worked hard to incorporate your feedback, and we hope that these revisions persuade you to accept our submission.

Sincerely,

Kengo Oka
