# Supplementary material for: Accuracy of AI-based binary classification for detecting malocclusion in the mixed dentition stage
Source: PLoS One. 2025 Dec 9;20(12):e0338713. doi: 10.1371/journal.pone.0338713 (PMC12688100; doi:10.1371/journal.pone.0338713)
Supplement: Attachment — Submitted filename: Response_to_Reviewers_auresp_2.docx [file pone.0338713.s002.docx]

**Response to Reviewers**

**Response to Academic editor:**

*Thank you for submitting your manuscript to PLOS ONE. After careful consideration, we feel that it has merit but does not fully meet PLOS ONE’s publication criteria as it currently stands. Therefore, we invite you to submit a revised version of the manuscript that addresses the points raised during the review process. Please thoroughly address the comments provided by Reviewers 2 & 4. Please submit your revised manuscript by Sep 13 2025 11:59PM. If you will need more time than this to complete your revisions, please reply to this message or contact the journal office at plosone@plos.org. When you're ready to submit your revision, log on to https://www.editorialmanager.com/pone/ and select the 'Submissions Needing Revision' folder to locate your manuscript file.*

***Please include the following items when submitting your revised manuscript:***

*・A rebuttal letter that responds to each point raised by the academic editor and reviewer(s). You should upload this letter as a separate file labeled 'Response to Reviewers'.*

*・A marked-up copy of your manuscript that highlights changes made to the original version. You should upload this as a separate file labeled 'Revised Manuscript with Track Changes'.*

*・An unmarked version of your revised paper without tracked changes. You should upload this as a separate file labeled 'Manuscript'.*

*If you would like to make changes to your financial disclosure, please include your updated statement in your cover letter. Guidelines for resubmitting your figure files are available below the reviewer comments at the end of this letter.*

**RESPONSE:**

We would like to express our sincere gratitude to the editors and reviewers for their valuable comments and suggestions. We have revised and improved the manuscript as you have indicated. Below are our responses to the issues raised. We have also highlighted in the “Revised Manuscript with Track Changes” file the areas where we have updated the manuscript in response to the reviewers' comments. Please note that there are no changes to the financial disclosure from the previous submission.

*If applicable, we recommend that you deposit your laboratory protocols in protocols.io to enhance the reproducibility of your results. Protocols.io assigns your protocol its own identifier (DOI) so that it can be cited independently in the future. For instructions see: https://journals.plos.org/plosone/s/submission-guidelines#loc-laboratory-protocols. Additionally, PLOS ONE offers an option for publishing peer-reviewed Lab Protocol articles, which describe protocols hosted on protocols.io. Read more information on sharing protocols at* [*https://plos.org/protocols?utm_medium=editorial-email&utm_source=authorletters&utm_campaign=protocols*](https://plos.org/protocols?utm_medium=editorial-email&utm_source=authorletters&utm_campaign=protocols)*.*

*We look forward to receiving your revised manuscript.*

**RESPONSE:**

Thank you for recommending that we register our protocol on Protocols.io. We hope to publish the results of our study as soon as possible in a peer-reviewed open access journal. However, the protocol registration process is expected to take some time. First, we need to prepare the necessary documents for protocol registration on Protocols.io. After that, we must obtain approval from the Institutional Review Board (IRB) regarding the publication of the protocol. Therefore, we will revisit this matter once the manuscript has been accepted for publication.

**Response to Reviewer #1:**

*Thank you for the opportunity to review the revised manuscript titled "Accuracy of AI-based binary classification for detecting malocclusion in the mixed dentition stage." The authors have thoroughly addressed the previous comments and substantially improved the manuscript in both content and clarity.*

*The rationale and relevance of the study are now well established, with improved contextualization of the epidemiology of malocclusion and the limitations of current screening tools. The methodological section has been clarified, particularly regarding the AI modeling process, dataset composition, and participant recruitment strategy. Importantly, the addition of precision and F1 scores strengthens the evaluation of the models given the potential for class imbalance.*

*The discussion now appropriately acknowledges the model's limitations, including the lack of external validation, potential device variability, and the non-diagnostic nature of the tool. The inclusion of logistic regression as a baseline was also a valuable addition.*

*Overall, the manuscript now meets the standards for publication. I recommend acceptance with minor revisions, limited to emphasizing in the conclusion that the AI model is intended as a screening tool and not a substitute for clinical diagnosis.*

**RESPONSE:**

We would like to express our gratitude to you for your insightful comments and suggestions, which have helped us improve our manuscript considerably. In conclusion section, we emphasized *that the AI model is intended as a screening tool and not a substitute for clinical diagnosis.* Our responses to the comments are provided below.

P3, lines 53 – lines 54

For the detection of malocclusion in mixed dentition, AI-based binary classification models are a promising approach as a screening tool.

P43, lines 747 – P44, lines 752

~~We used a smartphone application to collect dental images with aligned angles of view and used these images to build a binary classification model of malocclusion in the mixed dentition period using AI.~~

We used a smartphone application to collect dental images with aligned angles of view. Based on these images, we developed AI-based binary classification model for malocclusion during the mixed dentition period and verified its potential as a screening tool.

P44, lines 753 – P44, lines 757

~~Although further refinement is needed for clinical application, this study shows that the AI-based binary classification model is a promising approach to detect malocclusion in the mixed dentition period.~~

Further refinement is needed for clinical application, but this study shows that the AI-based binary classification model is a promising tool for screening malocclusion during the mixed dentition stage.

**Response to Reviewer #2:**

*I thank the authors for the clarifications provided in the revised manuscript. The use of smartphone images for orthodontic screening is indeed promising, and the study addresses a relevant and timely topic. That said, some important aspects still require clarification:*

**RESPONSE:**

We sincerely appreciate your insightful comments and suggestions, which have helped us to significantly improve our manuscript. Our responses to the comments are provided below.

***Introduction***

*The Introduction remains overly long, particularly with the additions made in the latest revision. I recommend streamlining this section to focus more directly on the study rationale and objectives.*

**RESPONSE:**

As you noted, the introduction was too long, so we updated it to emphasize the key points. Also, we deleted the redundant text. The revisions are as follows.

P4, lines 57 – lines 62

~~The prevalence of malocclusion is high worldwide, and its incidence rate remains at a high level from the deciduous dentition stage to the permanent dentition stage. Alhammadi et al. conducted a systematic review of 53 studies published by November 2016 that met the evaluation criteria and calculated the prevalence of malocclusion [1].~~

A systematic review by Alhammadi et al. revealed a high global prevalence of malocclusion [1].

P5, lines 73 – P6, lines 97

~~Castellote et al. found that the perception of malocclusion~~ ~~varies between individuals and between patients and practitioners [4]. Using frontal intraoral photographs of 24 cases classified by the DAI index, they examined the perception of dental aesthetics among dentists, orthodontists, and the general population. The study showed that there were significant differences in IOTN-AC scores between orthodontists, general dentists, and the general population, with orthodontists giving the most severe scores. This study also suggests that there may be a discrepancy between orthodontists and patients in their perception of the need for orthodontic treatment. In addition, Fujimura et al. compared the students' own perception of dental aesthetics and the results of the dentists' assessment using the IOTN-AC scale in 173 Japanese university students [5]. A comparison between the two showed a large discrepancy. Of the 33 students judged to need orthodontic treatment by the dentists, only 3 (9.1%) were judged to need orthodontic treatment by the students' self-assessment. These studies suggest that some patients themselves may not be aware that the degree of malocclusion is severe enough to require treatment.~~

Castellote et al. found that the perception of malocclusion varies among individuals, patients, and practitioners [4]. They examined the perception of dental aesthetics among orthodontists, general dentists, and the general population by using frontal intraoral photographs cases classified by the Dental Aesthetic Index (DAI). The results revealed significant differences in Index of Orthodontic Treatment Need-Aesthetic Component (IOTN-AC) scores, with orthodontists giving the most severe scores. Another study compared the self-perception of dental aesthetics among 173 Japanese university students with dentists' assessments using the IOTN-AC scale [5]. The comparison showed a large discrepancy: Of the 33 students judged by dentists to need treatment, only 3 (9.1%) believed they need it. These studies suggest that some patients may not be aware that their malocclusion is severe enough to require treatment.

P6, lines 106 – P7, lines 117

~~While patients visiting a dentist's office are diagnosed with malocclusion through a detailed examination such as head x-rays, in the field of health checkups and epidemiological studies,~~ ~~such as Dental Aesthetic Index (DAI) and Index of Orthodontic Treatment Need (IOTN) are used to detect malocclusion because precise examinations cannot be performed in the site. These are useful screening tools that quantify the need for orthodontic treatment by incorporating function, morphology, and dentition aesthetics into the evaluation criteria, but they are composed of many items and require a large amount of time for evaluation.~~

Malocclusion is diagnosed through detailed examinations, such as X-rays. However, in large-scale health checkups and epidemiological research, methods such as DAI and IOTN are used to screen for malocclusion. While these indices are useful for quantifying the need for orthodontic treatment, they are time-consuming and may result in inter-rater variability depending on the evaluator.

P7, lines 117 – lines 119

~~Also, it has been reported that the perception of malocclusion varies among evaluators and patients themselves~~ ~~[4].~~

P8, lines 127 – lines 138

~~Based on these reports, it is anticipated that algorithms can be developed to enable simple evaluation of oral health by maximizing the use of oral images and other machine learning methods such as CNN, thereby allowing patients to receive appropriate feedback regardless of location. Furthermore, by utilizing these algorithms as screening tools, it is expected that patients' awareness of visiting clinics will increase, enabling screening for oral diseases even in places where detailed examinations are difficult, such as health checkup venues or home.~~

Based on these reports, it is anticipated that we can develop algorithms for a simple oral health evaluation by using oral images and machine learning (e.g., CNN). This would not only provide patients with appropriate feedback regardless of their location but also serve as a screening tool to promote clinics visits and enable the detection of oral diseases in places where detailed examinations are difficult, such as at home or during health checkups.

***Data annotation process***

*The authors mention that three researchers jointly reviewed sample images of malocclusion and calibrated their judgments prior to the annotation process. While this is a positive step, the description remains vague and insufficiently detailed. To enhance transparency and reproducibility, I recommend that the authors clarify:*

*・How the calibration was conducted (e.g., through consensus rounds, training sessions, or use of reference cases).*

*・The specific diagnostic criteria used to define each type of malocclusion .*

**RESPONSE:**

Thank you for your insightful comments. We have provided detailed information on the data annotation process, including how calibration was performed. Furthermore, there was written mistake in the numerical values in the breakdown, so we have corrected them to the correct values. The corrections are as follows:

P15, lines 254 – 258

The criteria for annotation of each type of malocclusion are as follows:

Overbite: more than half of clinical crown in the lower front teeth are covered by the upper front teeth, Crowding: adjacent teeth overlap by more than 1/4 of the width of the crown, Overjet: overjet is large (approximately 4 mm or more), indicating protrusion of the upper front teeth.

P15, lines 259 – P16, lines 283

~~After that, three researchers simultaneously evaluated whether or not each subject had malocclusion. Before annotation, sample images of malocclusion were reviewed, and the three researchers calibrated their judgments before beginning annotation. Binary data on the presence and absence of dental malocclusion (overbite, overjet, and crowding) were obtained from smartphone dental images. Those with no clearly imaged dentition were excluded from the analysis before annotation. The breakdown of each malocclusion data after data annotation is shown below. overbite (n=166) / no overbite (n=342), overjet (n=147) / no overjet (n=368), crowding (n=166) / no crowding (n=342).~~

Based on the above criteria, three researchers annotated the data according to the following procedure. (1) The three researchers simultaneously reviewed reference case photos corresponding to each type of malocclusion (overbite, overjet, and crowding) and calibrated their judgments. (2) Based on each dental image dataset, each researcher independently annotated whether each subject had the corresponding type of malocclusion. (3) The three researchers simultaneously reviewed the annotation data assigned to each of them, and they adopted the value for cases where all three researchers made the same judgment. (4) When the three researchers had different opinions, they reviewed the reference case photos again and re-calibrated their judgments. (5) For subjects with differing judgments, each researcher each re-annotated the data. (6) The three researchers simultaneously reviewed the re-evaluated annotation data. Identical judgments were adopted, and cases with remaining discrepancies were deemed unevaluable and excluded from the dataset. The final breakdown of the dataset regarding the presence of malocclusion after data annotation is as follows: Dataset for overbite (n=508) : overbite (n=166) / no overbite (n=342), Dataset for overjet (n=515) : overjet (n=147) / no overjet (n=368), Dataset for crowding (n=518) : crowding (n=123) / no crowding (n=395).

*Whether inter- and/or intra-rater agreement was quantitatively assessed (e.g., Cohen’s kappa, intraclass correlation coefficients). If not, this limitation should be explicitly acknowledged in the Discussion section.*

**RESPONSE:**

Thank you for your valuable comments. This study did not quantitatively assess inter- or intra-rater agreement for data annotation. Therefore, we have mentioned this as a limitation discussed in the Discussion section. The revisions are detailed in the discussion section on the next page.

***Results – Table Footnote***

*Please consider removing the abbreviation “n.s.: not significant” from the table footnote, as this abbreviation does not appear to be used in the table itself.*

**RESPONSE:**

Thank you very much for pointing this out. We have removed this abbreviation from the footnotes in the table. We apologize for this written mistake.

***Discussion section***

*The two recently added paragraphs (“Third” and “Fourth”) regarding algorithm accuracy are somewhat repetitive. I suggest consolidating them to improve clarity and conciseness in the Discussion.*

**RESPONSE:**

Thank you for your insightful and valuable comments. As you pointed out, the discussion section contained repetitive wording about limitations for the model. We reviewed and consolidated the text again to make it more concise.

In the previous draft, the limitations of this study were listed in "First"and "Fourth" as challenges for improving accuracy. However, due to overlapping expressions, we have been consolidated into a single section. Similarly, "Second" and "Third" outlined challenges related to improving generalizability and real-world applicability. We have also been consolidated into a single section.

P39, lines 670 – 672

~~Therefore, in future studies, the dentition and occlusal status should be assessed by orthodontists, and these data should be used to develop an algorithm.~~

P42, lines 726 – P43, lines 732

~~Fourth, to verify the accuracy of the algorithm, it is necessary to compare the prediction results of the developed algorithm with the diagnostic values obtained by several orthodontists. In this study, we have not yet verified the extent to which the prediction values of the constructed algorithm agree with the diagnostic results of experienced orthodontists. In future research, it is important to compare the predictive accuracy of AI with the diagnostic accuracy of experienced orthodontists, construct a more reliable AI model, and further verify its clinical practicality.~~

P39, lines 672 – P40, lines 684

Additionally, this study did not quantitatively evaluate inter-rater and intra-rater agreement during assessment using metrics such as Cohen's kappa coefficient or intraclass correlation coefficient. In the future study, to enhance the algorithm's accuracy, it is crucial to adopt annotation judgment values provided by experienced orthodontists. Furthermore, it is critically important to build the algorithm based on annotation data demonstrating high inter-rater and/or intra-rater agreement. After constructing an algorithm using annotation data with higher accuracy for training, it is also necessary to compare the prediction results of the developed algorithm with the diagnostic values obtained by multiple orthodontists. This study has not yet verified the degree to which the predicted values from the constructed algorithm align with the diagnostic results of experienced orthodontists. Future research should compare the predictive accuracy of AI with the diagnostic accuracy of experienced orthodontists, build more reliable AI models, and further validate their clinical utility.

P40, lines 685 – P42, lines 725

~~Secondly, the dental images used in this study were collected by parents following the guiding frame of the application, but the angle of view may be slightly different due to manipulation by the parents themselves. Under real-world conditions, the angles of dental images taken may become even more diverse. Therefore, future studies should include images with different angles of view in the training dataset and evaluate the versatility of the system to see if it can make accurate judgments on a variety of images. Furthermore, to minimize the impact of the angle of view of the captured images, improvements to the application screen instructions and the UI design of the image capture screen are also considered necessary for the implementation of this algorithm. Third, in addition to the angle of view, it will be necessary to verify the versatility of the system in relation to differences in smartphone models and shooting environments. In this study, although we limited our analysis to participants who own smartphones running Android 6.0 or later or iOS 11.0 or later, and we excluded images that were too dark from the analysis, we did not restrict the brightness (Lux) of the lighting or the smartphone models. Therefore, we believe that we were able to collect images under a certain degree of diverse conditions. The AUC for three classification model exceeded 0.70 in 5-fold cross-validation and accuracy verification using a test dataset, suggesting that the model demonstrates consistent accuracy across diverse environments. However, it is necessary to verify whether similar results can be obtained with images taken using a wider range of smartphone models or older versions of smartphones. Furthermore, when implementing this model in an application, it is also important to implement logic to select input images, such as~~ ~~excluding overly dark images based on color values (e.g., RGB).~~

Second, this study has not sufficiently examined the applicability of data obtained in the real world. Under real-world conditions, the angles and quality of dental images taken may become even more diverse. Therefore, it is necessary to investigate how well performance is maintained when using photographs taken in the real world. This study employed a dataset of dental images captured without restrictions on location or ambient lighting, using only minimal dental guide frames and simple instructions. Furthermore, since dental images were collected from over 500 individuals in their home environments, we believe a certain level of diversity in the collected data was achieved. However, in real-world use, issues with angle diversity may arise even within the defined guide frame, depending on the parent's operational ability and IT literacy. Regarding image quality, while subjects were recruited using smartphones that were mainstream at the time of this study, significantly outdated or minor models were not evaluated. Therefore, in real-world scenarios, image input from a wider variety of devices is assumed. Moving forward, it is necessary to enhance the algorithm's versatility while simultaneously investigating its applicability in real-world scenarios. Moreover, implementing UI logic for selecting input images is crucial. This includes functions to exclude excessively dark images based on color values (e.g., RGB) or images that are significantly out of focus and unclear. Additionally, implementing an instruction interface that is easy for all subjects to understand is required.

P43, lines 732 – 733

~~Furthermore, external validation using an independent dataset is necessary.~~

Third, after constructing a classification model with enhanced accuracy and versatility, validation using external datasets must also be conducted in the future.

**Response to Reviewer #4:**

*The use of AI in detecting malocclusion is an interesting topic that warrants investigation. However, in my opinion, several elements of this work need to be improved or clarified.*

**Response:**

We sincerely appreciate the insightful your comments, we were able to make significant improvements to the manuscript. Our responses to the comments are as follows.

***Major concern:***

*1. Data Annotation, lines 222-33. It is mentioned that the images were annotated 'simultaneously' by three researchers. However, it is not clear if they annotated independently or together. Did they discuss? How did they reach the final conclusion when disagreement existed?*

**Response:**

Thank you very much for your valuable feedback. We have added detailed information regarding the data annotation process. This includes calibration and the methodology for each step leading to the final conclusion.

P15, lines 259 – P16, lines 283

~~After that, three researchers simultaneously evaluated whether or not each subject had malocclusion. Before annotation, sample images of malocclusion were reviewed, and the three researchers calibrated their judgments before beginning annotation. Binary data on the presence and absence of dental malocclusion (overbite, overjet, and crowding) were obtained from smartphone dental images. Those with no clearly imaged dentition were excluded from the analysis before annotation. The breakdown of each malocclusion data after data annotation is shown below. overbite (n=166) / no overbite (n=342), overjet (n=147) / no overjet (n=368), crowding (n=166) / no crowding (n=342).~~

Based on the above criteria, three researchers annotated the data according to the following procedure. (1) The three researchers simultaneously reviewed reference case photos corresponding to each type of malocclusion (overbite, overjet, and crowding) and calibrated their judgments. (2) Based on each dental image dataset, each researcher independently annotated whether each subject had the corresponding type of malocclusion. (3) The three researchers simultaneously reviewed the annotation data assigned to each of them, and they adopted the value for cases where all three researchers made the same judgment. (4) When the three researchers had different opinions, they re-examined the reference case photos and re-calibrated their judgments. (5) For subjects with differing judgments, each researcher each re-annotated the data. (6) The three researchers simultaneously reviewed the re-evaluated annotation data. Identical judgments were adopted, and cases with remaining discrepancies were deemed unevaluable and excluded from the dataset. The final breakdown of the dataset regarding the presence of malocclusion after data annotation is as follows: Dataset for overbite (n=508): overbite (n=166) / no overbite (n=342), Dataset for overjet (n=515): overjet (n=147) / no overjet (n=368), Dataset for crowding (n=518): crowding (n=123) / no crowding (n=395).

*2. Modeling and validation, lines 266-86. Although some details of the algorithms used were provided after the first revision, it is unclear why different methods/ models were applied to different conditions (overbite, overjet, crowding). Clarifications are needed.*

**Response:**

Thank you for your valuable comments. This study used Datarobot for algorithm construction. This tool enables the simultaneous construction of multiple robust ensemble models through its Autopilot feature. Due to the tool's specifications, DataRobot prioritizes higher accuracy over simpler model structures. Consequently, the classification models obtained in this study (overbite, overjet, crowding) exhibit significantly different structures. In response to your insightful feedback, we have added supplementary explanations regarding DataRobot's characteristics and modeling strategy.

P14, lines 246 – P15, lines 249

Note that due to the tool's specifications, DataRobot prioritizes higher accuracy over simpler model structures. Therefore, this study did not evaluate structural similarity between models and instead focused on models that demonstrated superior accuracy.

*3. Related to the above concern, since the predicted values using the image features from Elastic Net Classifiers were used as explanatory variables for overjet and crowding, would this have impacted the importance of the images? A comparison between this model and a model using the image features (instead of predicted values from the image features) may ease this concern.*

**Response:**

Thank you very much for your insightful comments. In this study, Datarobot treated each input variable as a single column (with each dental image as one row) to calculate the PI score. PI score indicates how much the model's performance degrades when DataRobot randomly shuffles the elements of an input variable (a specific column) within the model (while leaving other columns unchanged). Therefore, we believe the interpretation of the importance of each explanatory variable in the model decision-making process, as shown in this paper, remains unchanged. That is, even when adopting a stacked model structure, the contribution of the image within that model is not overestimated. While comparing PI values between models is difficult due to structural differences among the models constructed this time, we consider the interpretation of PI within each constructed model to be consistent.

*4. The values of conducting both chi-sq test (lines 340 - 48) and the logistic regression (lines 328-337) are unclear. In fact, chi-sq test can only check the association between the response variable and one single explanatory variable (Q1 - Q4) at a time, while logistic regression can assess the relationship between the response and all explanatory variables. From the logistic regression model, adjusted odds ratios can be obtained as well. In my opinion, there is no need to do both.*

**Response:**

Thank you for your valuable feedback and we apologize for any confusion. Taking into account another reviewer's comments, we included logistic regression as a baseline for comparison with the machine learning model constructed in this study.

Furthermore, as shown in Figure 1, the dataset used for the chi-square test, which examined the association between malocclusion and oral habits, differs from the dataset used to construct the machine learning model.

For the machine learning process, we further cleaned the data from the dataset used for the chi-square test. For the logistic regression model in this study, we used the same dataset (only questionnaire) as when building the machine learning model to enable direct comparison. To avoid ambiguity, we emphasized the difference in datasets as follows.

P23, lines 393 – 395

Furthermore, to enable comparison with machine learning models and their accuracy, this analysis employed the same dataset used for the machine learning models.

P23, lines 404 – 406

As shown in Fig. 1, this analysis was performed using the dataset after data annotation (prior to data cleansing for machine learning model construction).

*5. Lines 410-33. In presenting the results, there is no need to mention every metric.*

**Response:** Thank you for your comments. In this results section, we have limited our presentation to key indicators only.

We have made the following corrections:

P27, lines 477 – P28, lines 482

The three machine learning models showed high performance, with an AUC of 0.70 or higher, which is an overall accuracy metric (Table 2). The overbite model maintained high performance in detecting overbite, with an AUC of 0.92, sensitivity of 91.2%, specificity of 81.4%~~, accuracy of 85.0%, precision of 73.8%, and F1 score of 0.8158~~. The overjet model showed moderate accuracy, with an AUC of 0.74, sensitivity of 70.0%, specificity of 71.2%~~, and accuracy of 71.0%, precision of 40.0%, and F1 score of 0.5091~~. The crowding model also showed moderate accuracy, with an AUC of 0.73, sensitivity of 80.0%, specificity of 61.6%~~, and accuracy of 66.3%, precision of 41.7%, and F1 score of 0.5479~~. The other metric were shown in Table 2.

P28, lines 489 – 496

The three logistic regression models showed low performance with an AUC of less than 0.70 (Table 3). The overbite model showed low performance, with an AUC of 0.52, sensitivity of 100.0%, specificity of 0.0%~~, accuracy of 36.6%, precision of 36.6%, and F1 score of 0.5354.~~ The overjet model also showed low accuracy, with an AUC of 0.55, sensitivity of 100.0%, specificity of 4.1%~~, and accuracy of 24.7%, precision of 22.2%, and F1 score of 0.3636~~. The crowding model also showed low accuracy, with an AUC of 0.62, sensitivity of 48.0%, specificity of 75.3%~~, and accuracy of 68.4%, precision of 40.0%, and F1 score of 0.4364~~. The other metric were shown in Table 3.

*6. Lines 438-47. The associations between the presence of conditions and the questionnaire questions are important. More details should be provided. For example, instead of 'there is a significant association', more details can be provided - whether some habits increase/ reduce the risk of developing a certain condition?*

**Response:** Thank you for your valuable feedback, we have provided more detailed results indicating whether particular oral habits pose a high or low risk of malocclusion. The revisions are as follows:

P29, lines 502 – P30, lines 520

~~However, a significant association was observed between the presence or absence of overjet and questionnaire items on habits related to incompetent lip seal (Question 1: Is your child’s mouth often open during the day? Question 2: Does your child sleep with their mouth open?) (~~*~~P~~* ~~<0.001 and~~ *~~P~~* ~~< 0.05, OR = 2.16 and 1.60, respectively) (Table 5). Furthermore, a significant association was observed between questionnaire items on the habit of eating hard foods (Question 3: Does your child have difficulty eating hard foods? Question 4: Does your child often prefer soft foods?) and the presence or absence of crowding (~~*~~P~~* ~~< 0.01, OR = 1.93 and 1.82, respectively) (Table 6).~~

However, oral habits related to incompetent lip seal (Question 1: Is your child’s mouth often open during the day? Question 2: Does your child sleep with their mouth open?) recorded significantly higher frequency in people with overjet than in people with no overjet (P <0.001 and P < 0.05, OR = 2.16 and 1.60, respectively) (Table 5). Incompetent lip seal was suggested to be a risk factor for overjet. Furthermore, oral habits related to avoiding hard foods (Question 3: Does your child have difficulty eating hard foods? Question 4: Does your child often prefer soft foods?) recorded significantly higher frequency in people with crowding than in people with no crowding (P < 0.01, OR = 1.93 and 1.83, respectively) (Table 6). The result showed that oral habits related to avoiding hard foods to be a risk factor for crowding.

***Minor comments:***

*1. Lines 34 - meaning of 'Q1-Q4' is not clear in the abstract. Suggest replacing with some simple descriptions.*

**Response:** Thank you for your comments. We have added a simple discription of these questions. The revisions are as follows:

P3, lines 41 – 42

Three dental images were used for all model building, and questionnaire data used all four questions ~~(Q1–Q4)~~ about oral habits (Q1: mouth open during the day, Q2: sleep with mouth open, Q3: have difficulty eating hard foods, Q4: prefer soft foods) for the overbite classification model, Q1 and Q3 for the overjet classification model, and Q1 and Q4 for the crowding classification model.

*2. Lines 163-4 - clarify if the participant was 'dropped out' or 'excluded'.*

**Response:** We apologized for the mistake, and the correct expression is “excluded.”

The revision is as follows. Similarly, in Fig. 1, the term “dropped out” was used, so it has been revised to “excluded.”

P11, lines 191 – 192

During this study, 1 participant ~~dropped out~~ was excluded (not meeting the inclusion criteria).

*3. Lines 240-44. These sentences seem to repeat the previous ones.*

**Response:** Thank you for pointing that out. Since there were duplicated sections, we have consolidated these passages.

P17, lines 286 – 298

~~If at least one of the images of the three teeth in the anterior region, left side, or right side of the dentition was not taken at the central occlusion position, or if the anterior teeth of the mandible were not properly captured, the subject was excluded from the dataset used to construct the algorithm. For dental images that were not in the central occlusal position, the images were not adopted in the training dataset for overbite or overjet classification model. Additionally, if the mandibular anterior teeth were not adequately captured due to overbite or other reasons, and the images were not adopted in the training dataset for crowding classification model.~~

Subjects for whom at least one of the three dental images was not captured in the central occlusion position were excluded from the training dataset used to develop the overbite and overjet classification models. Furthermore, data from subjects whose mandibular anterior teeth were inadequately captured in any of the three images due to overbite or other reasons were excluded from the training dataset for the dental crowding classification model.

*4. Lines 244-47. Rewrite this sentence.*

**Response:** Thank you for your comments. To avoid ambiguity, we have revised the text as follows.

~~The breakdown of each malocclusion data after data cleansing for machine learning is shown below(overbite (n=155) / no overbite (n=308), overjet (n=138) / no overjet (n=329), crowding (n=112) / no crowding (n=381)) .~~

P18, lines 302 – 305

The final breakdown of the dataset regarding the presence of malocclusion after data cleansing is as follows:

Dataset for overbite (n=463): overbite (n=155) / no overbite (n=308), Dataset for overjet (n=467): overjet (n=138) / no overjet (n=329), Dataset for crowding (n=493): crowding (n=112) / no crowding (n=381).

Additionally, the breakdown of data after annotation has been revised for clarity.

P16, lines 279 – 283

~~The breakdown of each malocclusion data after data annotation is shown below. overbite (n=166) / no overbite (n=342), overjet (n=147) / no overjet (n=368), crowding (n=166) / no crowding (n=342).~~

The final breakdown of the dataset regarding the presence of malocclusion after data annotation is as follows:

Dataset for overbite (n=508): overbite (n=166) / no overbite (n=342), Dataset for overjet (n=515): overjet (n=147) / no overjet (n=368), Dataset for crowding (n=518): crowding (n=123) / no crowding (n=395).

*5. Line 256. Either "...used in the algorithm" or "...used in the analysis", but not "algorithm analysis"*

**Response:** Thank you for your comment, we have revised the text as follows.

P18, lines 311 – 314

The categorical variable, the presence or absence of the corresponding dentition type, was quantified as 1 for “applicable” and 0 for “not applicable,” and the questionnaire response results were quantified as 1 for “yes” and 0 for “no” and used in the algorithm ~~analysis~~.

*6. Line 266. "Using the training dataset", not "80% of the training dataset". Similar in Line 313.*

**Response:** We applogized for this mistake. we have revised the text as follows.

P19, lines 324 – 325

Using ~~80% of~~ the training dataset, three algorithms were constructed to determine the presence of overbite, ~

P21, lines 373 – P22, lines 374

The ~~remaining 20% of the~~ validation dataset was used to evaluate the generalization performance of new data in the constructed model.

*7. Line 286. "classification"*

**Response:** Thank you for your feedback, We have corrected “classgfcation” to “classification”.

P20, lines 342 – 345

By feeding the binary data from the questionnaire and the prediction values into the SVM Classifier model as explanatory variables, we constructed a crowding ~~classgfcation~~ classification model.

*8. Line 292. "select" instead of "selected"*

**Response:** Thank you for your feedback, We have corrected it as below:

P20, lines 351 – 353

We considered inappropriate models and didn't select~~ed~~ models that only focus on other areas such as fingers instead of the dental area in the Activation map even if the AUC value is high.

**Response to Editor’s Email**

*While revising your submission, please upload your figure files to the Preflight Analysis and Conversion Engine (PACE) digital diagnostic tool, https://pacev2.apexcovantage.com/. PACE helps ensure that figures meet PLOS requirements. To use PACE, you must first register as a user. Registration is free. Then, login and navigate to the UPLOAD tab, where you will find detailed instructions on how to use the tool. If you encounter any issues or have any questions when using PACE, please email PLOS at figures@plos.org. Please note that Supporting Information files do not need this step.*

**RESPONSE:** Following the instructions, we have confirmed using PACE that the figures meet the requirements of PLOS One.

Thank you again for giving us the opportunity to improve our manuscript with your insightful comments. We have worked hard to incorporate your feedback, and we hope that these revisions persuade you to accept our submission.

Sincerely,

Kengo Oka
