# Supplementary material for: Accuracy of AI-based binary classification for detecting malocclusion in the mixed dentition stage
Source: PLoS One. 2025 Dec 9;20(12):e0338713. doi: 10.1371/journal.pone.0338713 (PMC12688100; doi:10.1371/journal.pone.0338713)
Supplement: Attachment — Submitted filename: Response_to_Reviewers_auresp_3.docx [file pone.0338713.s003.docx]

**Response to Reviewers**

**Response to Academic editor:**

*Thank you for submitting your manuscript to PLOS ONE. After careful consideration, we feel that it has merit but does not fully meet PLOS ONE’s publication criteria as it currently stands. Therefore, we invite you to submit a revised version of the manuscript that addresses the points raised during the review process. In addition to fixing the typos and presenting format, please address Reviewer 5's comments on the diagnostic criteria, inclusion/exclusion criteria, and limitations of detection accuracy. Please submit your revised manuscript by Nov 14 2025 11:59PM. If you will need more time than this to complete your revisions, please reply to this message or contact the journal office at* [*plosone@plos.org*](mailto:plosone@plos.org)*. When you're ready to submit your revision, log on to* [*https://www.editorialmanager.com/pone/*](https://www.editorialmanager.com/pone/) *and select the 'Submissions Needing Revision' folder to locate your manuscript file.*

***Please include the following items when submitting your revised manuscript:***

- *A rebuttal letter that responds to each point raised by the academic editor and reviewer(s). You should upload this letter as a separate file labeled 'Response to Reviewers'.*
- *A marked-up copy of your manuscript that highlights changes made to the original version. You should upload this as a separate file labeled 'Revised Manuscript with Track Changes'.*
- *An unmarked version of your revised paper without tracked changes. You should upload this as a separate file labeled 'Manuscript'.*

*If you would like to make changes to your financial disclosure, please include your updated statement in your cover letter. Guidelines for resubmitting your figure files are available below the reviewer comments at the end of this letter.*

**RESPONSE:**

We would like to express our sincere gratitude to the editors and reviewers for their valuable comments and suggestions. We have revised and improved the manuscript as you have indicated. Below are our responses to the issues raised. We have also highlighted in the “Revised Manuscript with Track Changes” file the areas where we have updated the manuscript in response to the reviewers' comments. Please note that there are no changes to the financial disclosure from the previous submission.

*If applicable, we recommend that you deposit your laboratory protocols in protocols.io to enhance the reproducibility of your results. Protocols.io assigns your protocol its own identifier (DOI) so that it can be cited independently in the future. For instructions see: https://journals.plos.org/plosone/s/submission-guidelines#loc-laboratory-protocols. Additionally, PLOS ONE offers an option for publishing peer-reviewed Lab Protocol articles, which describe protocols hosted on protocols.io. Read more information on sharing protocols at* [*https://plos.org/protocols?utm_medium=editorial-email&utm_source=authorletters&utm_campaign=protocols*](https://plos.org/protocols?utm_medium=editorial-email&utm_source=authorletters&utm_campaign=protocols)*.　We look forward to receiving your revised manuscript.*

**RESPONSE:**

Thank you for recommending that we register our protocol on Protocols.io. We hope to publish the results of our study as soon as possible in a peer-reviewed open access journal. However, the protocol registration process is expected to take some time. First, we need to prepare the necessary documents for protocol registration on Protocols.io. After that, we must obtain approval from the Institutional Review Board (IRB) regarding the publication of the protocol. Therefore, we will revisit this matter once the manuscript has been accepted for publication.

**Response to Reviewer #4:**

*Thank you for putting the effort in revising this manuscript. I believe the paper can be accepted after a careful proofread. Some suggestions/ grammatical errors I spotted include:*

*・Lines 256-258: 'Dataset' should not be capitalized after a comma.*

*・Line 274: suggest removing 'for new data'. Same for line 349.
・In the results section, suggest not including the whole questionnaire questions to improve readability (e.g.,*

*in lines 451-452, simply 'Questions 1 and 2' would be sufficient.*

**RESPONSE:**

We would like to express our gratitude to you for your insightful comments and suggestions, which have helped us improve our manuscript considerably. We have made the following corrections to the suggestions and grammatical errors you pointed out.

P14, lines 246 – lines 249

Dataset for deep bite (n=508): deep bite (n=166) / no deep bite (n=342), ~~D~~dataset for maxillary protrusion (n=515): maxillary protrusion (n=147) / no maxillary protrusion (n=368), ~~D~~dataset for crowding (n=518): crowding (n=123) / no crowding (n=395).

P15, lines 264 – lines 267

Dataset for deep bite (n=463): deep bite (n=155) / no deep bite (n=308), ~~D~~dataset for maxillary protrusion (n=467): maxillary protrusion (n=138) / no maxillary protrusion (n=329), ~~D~~dataset for crowding (n=493): crowding (n=112) / no crowding (n=381).

P16, lines 282 – lines 284

Of the total data, 80% was randomly split as a training dataset for algorithm construction and the remaining 20% as a validation dataset ~~for new data~~.

P21, lines 358 – lines 360

We used 80% of the data as a training dataset for model construction and the remaining 20% as a validation dataset ~~for new data~~.

P24, lines 411 – lines 416

The frontal image (PI: 100%) was the most important explanatory variable in the model, followed by the left side image (PI: 91.4%), right side image (PI: 64.7%), questionnaire data on habits related to incompetent lip seal (Question 1~~Q1: Is your child’s mouth often open during the day?)~~ (PI: 19.0%), and questionnaire data on the habit of eating hard foods (Question 3~~Q3: Does your child have difficulty eating hard foods)~~ (PI: 4.21%) (Fig 5a).

P25, lines 427 – lines 430

The frontal image (PI: 100%) was the most important explanatory variable in the model, followed by the right side image (PI: 2.32%) and questionnaire data on the habit of eating hard foods (Question 4~~Q4: Does your child often prefer soft foods?~~) (PI: 2.01%) (Fig 6a)

P28, lines 471 – lines 473

However, oral habits related to incompetent lip seal (Questions 1 and 2~~: Is your child’s mouth often open during the day? Question 2: Does your child sleep with their mouth open?~~) recorded significantly ~

P28, lines 477 – PY, lines 479

Furthermore, oral habits related to avoiding hard foods (Questions 3 and 4~~: Does your child have difficulty eating hard foods? Question 4: Does your child often prefer soft foods?~~) recorded significantly higher frequency ~

**Response to Reviewer #5:**

*This is an interesting study that explores the use of artificial intelligence (AI) to detect malocclusion in children during the mixed dentition phase, based on three intraoral photographs (frontal, left, and right views) taken by parents. While the concept is promising and relevant, there are several important issues that need to be addressed:*

**RESPONSE:**

We sincerely appreciate your insightful comments and suggestions, which have helped us to significantly improve our manuscript. Our responses to the comments are provided below.

*1. Figure Legends: The figure legends are embedded directly within the main text. To my knowledge, most academic journals typically require figure legends to be placed in a separate section at the end of the manuscript, not within the main body. I recommend the authors double-check the journal’s formatting guidelines and revise accordingly.*

*2. Tables:
Similarly, the tables are also embedded within the main text. Again, it is unclear whether PLOS ONE allows this format. Typically, tables should be presented separately, often on individual pages at the end of the manuscript. Please confirm with the journal’s formatting requirements and revise if necessary.*

**RESPONSE:**

Thank you for your comments. Regarding figure and table descriptions, we have placed them in the paragraph immediately following the citation within the main text, in accordance with the PLOS ONE guidelines. Therefore, we will maintain the current format. We have also reviewed the PLOS ONE formatting guidelines again to confirm they are correct.

*3. Diagnostic Criteria: On page 13, the authors state: “The criteria for annotation of each type of malocclusion are as follows: Overbite: more than half of clinical crown in the lower front teeth are covered by the upper front teeth, Crowding: adjacent teeth overlap by more than 1/4 of the width of the crown, Overjet: overjet is large (approximately 4 mm or more), indicating protrusion of the upper front teeth.” These criteria seem to lack comprehensiveness. What about cases of open bite or anterior crossbite? These types of malocclusion also require treatment and may be even more clinically significant than deep overbite or excessive overjet. The current classification system used by the authors may exclude these important cases, which represents a major methodological limitation.*

**RESPONSE:**

Thank you for your insightful comments. This study similarly evaluated the presence of other malocclusions important from a dental perspective—such as open bite, crossbite, mandibular protrusion etc. —in addition to maxillary protrusion, crowding, and deep bite. The presence of these malocclusions was determined based on the School Dentist Guidelines and prior studies referenced in the text. In this study, we comprehensively annotated the presence of each malocclusion. Among these, we focused on deep overbite, maxillary protrusion, and crowding to construct three binary classification models.

For example, a case annotated as “0” for maxillary protrusion indicates the absence of maxillary protrusion according to the above criteria. This does not necessarily imply the absence of other malocclusions (a case annotated as “0” for maxillary protrusion may still be annotated as “1” for crossbite, for example). Furthermore, even cases annotated as “1” for maxillary protrusion do not imply the absence of other malocclusions and may include coexisting conditions such as deep bite. Therefore, both the “0” and “1” datasets used in the binary classification model constructed here contain diverse dental data. Within this diverse dataset, we developed algorithm to determine the presence or absence of specific types of malocclusion. To clarify, we have made the following corrections.

P13, lines 223 – lines 231

The dentition and occlusal status of children were comprehensively determined for the presence of each type of malocclusion

from smartphone dental images based on the School Dentist Guidelines of the Japan Association of School Dentists and previous study [13]. ~~The criteria for annotation of each type of malocclusion~~ The annotation criteria for each malocclusion type (deep bite, maxillary protrusion, crowding) used in constructing the three algorithms in this study are as follows: Deep bite: more than half of clinical crown in the lower front teeth are covered by the upper front teeth, crowding: adjacent teeth overlap by more than 1/4 of the width of the crown, maxillary protrusion: overjet is large (approximately 4 mm or more), indicating protrusion of the upper front teeth.

As you pointed out, dental treatment is extremely important for conditions such as open bite, and we believe the development of screening tools for these conditions is also necessary. Moving forward, we plan to conduct research aimed at constructing algorithms capable of binary classification for conditions such as open bite, crossbite, mandibular protrusion, and other malocclusions. In Japan, the prevalence of these malocclusions tends to be lower compared to dental crowding or maxillary protrusion. Among the 519 subjects recruited for this study, only a portion exhibited these specific dental irregularities. When constructing algorithms and performing machine learning, it is necessary to secure a sufficient number of cases with specific malocclusion types. For malocclusions like open bite and crossbite, we need to collect more cases to create the algorithm. This time, as feasibility study, we focused on major malocclusions with higher prevalence rates and explored algorithm construction. We plan to develop a classification algorithm capable of handling all types of malocclusion in the future. Furthermore, we intend to build an ensemble model where algorithms for binary classification of each malocclusion type are connected in parallel. This ensemble model would enable simultaneous risk assessment for multiple malocclusion types with a single input of images and questionnaires.

*4. Terminology – “No Overjet” / “No Overbite”:
On page 14, the authors write: “Dataset for overbite (n=508): overbite (n=166) / no overbite (n=342), Dataset for overjet (n=515): overjet (n=147) / no overjet (n=368), Dataset for crowding (n=518): crowding (n=123) / no crowding (n=395).”
The repeated use of expressions such as “no overjet” and “no overbite” is problematic. Overjet and overbite are quantitative features; it is not accurate to say a person has “no overjet” since everyone has some degree of overjet, whether it is positive or negative. It would be more appropriate to use terms such as “normal overjet” vs. “abnormal overjet,” and similarly for overbite. The manuscript contains many such instances that need to be revised.*
**RESPONSE:**

We sincerely apologize for any confusion caused by the incorrect expression. Following the criteria outlined in the main text, we have classified these into two categories: large overjet or no large overjet, as well as large overbite or no large overbite. As you pointed out, to avoid confusion, we have revised the terminology in the main text as follows: “overjet” has been changed to “maxillary protrusion,” and ‘overbite’ has been changed to “deep bite.” Furthermore, in line with the terminology changes, we have revised not only the main text but also the terminology used in Figure 1.

*Moreover, even if the terminology is corrected, it still does not address the concern raised in point 3. What about cases with insufficient overbite or overjet? Based on the current dataset, it seems the authors have only included cases with excessive overjet or deep bite. This makes the scope of the AI detection system quite narrow. How would the model perform with open bite or anterior crossbite cases? Would the algorithm still be effective?*

**RESPONSE:**

Thank you for your insightful comments. We have collected a diverse range of dental aligment, we are not solely collecting cases with excessive overbites or perfectly aligned teeth. This model can reliably determine the presence or absence of crowding, maxillary protrusion, and deep overbite even in cases presenting with open bite or anterior crossbite. As mentioned earlier, for each case, we comprehensively assessed various malocclusions (including open bite, crossbite, and mandibular protrusion etc.). For each subject, we annotated the presence or absence of multiple types of malocclusion. For example, for the maxillary protrusion dataset, we constructed the dataset by sorting it into 0/1 based on whether maxillary protrusion was present or absent. This means that since other malocclusion types were also assessed, a person labeled 0 for maxillary protrusion does not necessarily mean they have no other malocclusions, and a person labeled 1 does not mean they only have maxillary protrusion. Thus, both the 0 and 1 sides of the constructed dataset are rich in diverse dental alignment. Since the learning model was built on this foundation, it can detect other malocclusions even when present, making this classification algorithm versatile.

This study demonstrates the potential and usefulness of algorithms utilizing AI and smartphone images; it does not represent a finished product ready for implementation. We currently plan to gather more cases to build classification models for each malocclusion type( include open bite, crossbite, etc.). By constructing binary classification models for all types of dental malocclusion and connecting these multiple algorithms in parallel, it will be possible to assess the risk for each malocclusion type from a single data input. This will enable us to implement a screening tool for risk assessment across all possible dental malocclusion.

*5. Presentation of Demographic Data:
On pages 21–22, the section describing “Residential area” and “Annual household income” is written in incomplete sentences and lacks clarity. Including this in the main body of the text in its current form is awkward. It may be better to present this data using a figure or a well-formatted table to improve readability.*

**RESPONSE:**

Thank you for your valuable comments. Based on your feedback, we created a table to improve readability. We created a table showing the residential areas of the 519 participants. Furthermore, using data from a total of 447 participants—excluding the 72 who did not answer the household income question—we created a table regarding household income.

P22, lines 377 – lines 382

The following is a breakdown of the residential areas ~~and annual household incomes~~ of the 519 participants recruited in this study~~children recruited this time~~ (Table 2). ~~Residential area: Hokkaido area 4.43%, Tohoku area 4.82%, Kanto area 32.2%, Chubu area 19.1%, Kinki area: 20.0%, Chugoku area:5.97%, Shikoku area:2.89%, Kyusyu area:10.6%.~~

**Table 2. Breakdown of participants by residential area.**

| Residential area | Number of participants (*n*) | Percentage of participants (%) |
| --- | --- | --- |
| Hokkaido | 23 | 4.4 |
| Tohoku | 25 | 4.8 |
| Kanto | 167 | 32.2 |
| Chubu | 99 | 19.1 |
| Kinki | 104 | 20.0 |
| Chugoku | 31 | 6.0 |
| Shikoku | 15 | 2.9 |
| Kyusyu | 55 | 10.6 |

P22, lines 383 – lines 391

Of the 519 participants in this study, the breakdown of household annual income for the total of 447 participants who answered the household income question is shown below (Table 3).

~~Annual household income: under than 2 million yen: 10.5%, 2 to under 4 million yen: 25.7%, 4 to under 6 million yen: 26.9%, 6 to under 8 million yen: 17.5%, 8 to under 10 million yen: 9.9%, 10 to under 12 million yen : 4.9%, 12 to under 15 million yen: 2.5%, 15 to under 20 million yen: 1.2%, 20 or more million yen: 0.8%, no answer: 13.9%.~~

**Table 3. Breakdown of participants by annual household income.**

| Annual household income (yen) | Number of participants (*n*) | Percentage of participants (%) |
| --- | --- | --- |
| under than 2 million | 15 | 3.4 |
| 2 to under 4 million | 68 | 15.2 |
| 4 to under 6 million | 148 | 33.1 |
| 6 to under 8 million | 116 | 26.0 |
| 8 to under 10 million | 59 | 13.2 |
| 10 to under 12 million | 27 | 6.0 |
| 12 to under 15 million | 9 | 2.0 |
| 15 to under 20 million | 4 | 0.9 |
| 20 or more million yen | 1 | 0.2 |

*6. Inclusion/Exclusion Criteria – Figure 3(c):
In Figure 3(c), the child appears to be biting towards the right side with a slight forward posture. According to the authors’ own inclusion/exclusion criteria, this case should have been excluded. This raises concerns about the consistency in applying the inclusion criteria.*

**RESPONSE:**

Thank you for your valuable comments. This time, when constructing machine learning models for maxillary protrusion and deep bite, we excluded cases clearly judged not to be in centric occlusion. Specifically, we excluded cases where a gap was clearly visible (e.g., the tongue was visible through the gap), the occlusal relationship did not match between images (e.g., normal overbite in the frontal view but reversed occlusion only in the right dental image). Therefore, the annotation criteria at that time were consistent among researchers. Furthermore, upon re-examining images excluded due to annotations indicating they clearly were not in centric occlusion, all met the aforementioned criteria (presence of obvious gaps, inconsistent occlusal relationships across the three images). Additionally, there was no disagreement among researchers regarding the judgment values for whether the position was clearly not in centric occlusion based on the above criteria, demonstrating consistency throughout this study.

While standardizing the shooting angle on the application screen, slight variations may occur in images taken by parents due to operating methods or the child's irregular movements. Therefore, the range of acceptable images must allow for redundancy and versatility. This algorithm is intended as a screening tool or an awareness-raising tool to encourage visits to dental clinics, not for precise diagnosis. Therefore, images like those presented here were also accepted and used for training. To avoid misunderstanding, the annotation method description has been revised as follows.

P15, lines 255 – lines 260

~~Subjects for whom at least one of the three dental images was not captured in the central occlusion position were excluded from the training dataset used to develop the deep bite and maxillary protrusion classification models.~~

Subjects for whom at least one of the three dental images were clearly not taken in centric occlusion (e.g., cases where gaps were clearly visible, such as the tongue being visible through space, or where the occlusal relationship did not match between images, etc.) were excluded from the training dataset used to develop the classification models for deep bite and maxillary protrusion.

*7. Limitations of Detection Accuracy:
The manuscript reports that: “The overjet and crowding classification models showed moderate accuracy (AUC > 0.70).”　This result is somewhat expected. Since the photographs provided by parents are limited to frontal, left, and right views, there are inherent limitations in identifying certain conditions, especially crowding. For example, in deep bite cases, the lower anterior teeth may be obscured in frontal and lateral photos, making it difficult for AI to detect crowding in the lower arch. A photo taken with occlusal view would be far more informative for evaluating crowding, but it is not provided in this study.*

**RESPONSE:**

Thank you for your insightful comments. The use of occlusal photographs may warrant consideration in the future; however, it is considered extremely difficult for parents to capture clear images of the occlusal surfaces using smartphones. The primary purpose of this model is to create a simple screening tool utilizing images that parents can capture and questions designed to encourage dental visits; it is not intended for precise diagnosis. Therefore, occlusal photographs were not incorporated into the model construction in this study.

Furthermore, as described in the Methods section, when deep overbite causes excessive concealment of the entire mandibular dentition, it is impossible to evaluate the presence or absence of dental crowding in that region. Therefore, such cases have been excluded from the dental crowding classification model dataset. Furthermore, following comments from a previous reviewer, we have emphasized in the introduction and discussion sections that this tool is a screening tool based on data obtained from parents and is not a precise medical diagnosis. As you all pointed out, we consider this algorithm to be positioned as a screening tool, not a sophisticated diagnostic tool.

**Response to Editor’s Email**

*While revising your submission, please upload your figure files to the Preflight Analysis and Conversion Engine (PACE) digital diagnostic tool, https://pacev2.apexcovantage.com/. PACE helps ensure that figures meet PLOS requirements. To use PACE, you must first register as a user. Registration is free. Then, login and navigate to the UPLOAD tab, where you will find detailed instructions on how to use the tool. If you encounter any issues or have any questions when using PACE, please email PLOS at figures@plos.org. Please note that Supporting Information files do not need this step.*

**RESPONSE:** Following the instructions, we have confirmed using PACE that the figures meet the requirements of PLOS One.

Thank you again for giving us the opportunity to improve our manuscript with your insightful comments. We have worked hard to incorporate your feedback, and we hope that these revisions persuade you to accept our submission.

Sincerely,

Kengo Oka
